# Epidemiology, clinical and pathological features and outcomes of listeriosis in ruminants: a systematic review and meta-analysis

**DOI:** 10.1080/01652176.2025.2598257

**Published:** 2025-12-08

**Authors:** Inmaculada López-Almela, Chirag C. Sheth, Jesús Gomis, Ángel Gómez-Martín, Marc Lecuit, Juan J. Quereda

**Affiliations:** a Research Group Listeria: Biology and Infection. Departamento Producción y Sanidad Animal, Salud Pública Veterinaria y Ciencia y Tecnología de los Alimentos, Facultad de Veterinaria, Universidad Cardenal Herrera-CEU, CEU Universities, Valencia, Spain; b Faculty of Health Sciences, Universidad Cardenal Herrera-CEU, CEU Universities, Valencia, Spain; c Research Group Microbiological Agents Associated with Animal Reproduction (ProVaginBIO). Departamento Producción y Sanidad Animal, Salud Pública Veterinaria y Ciencia y Tecnología de los Alimentos, Facultad de Veterinaria, Universidad Cardenal Herrera-CEU, CEU Universities, Valencia, Spain; d Institut Pasteur, Université Paris Cité, Inserm U1117, Biology of Infection Unit, Paris, France; e Institut Pasteur, Listeria National Reference Centre and WHO Collaborating Centre, Paris, France; f Division of Infectious Diseases and Tropical Medicine, Institut Imagine, APHP, Necker-Enfants Malades University Hospital, Paris, France

**Keywords:** Cattle, sheep, goat, infection, morbidity, case fatality rate, antibiotic treatment, gross lesions, histopathology

## Abstract

We performed a systematic review and meta-analysis of the epidemiology, clinical and pathological features, outcomes, and therapy for listeriosis in ruminants. PubMed, Web of Science, and Scopus were searched with no publication date limits. A random-effects meta-analysis model was used to calculate the pooled effect size using morbidity and case fatality rate data. 63 and 38 studies met the inclusion criteria for the systematic review and meta-analysis, respectively. 56 out of 63 studies were published before 2016 when cgMLST was developed. A comprehensive analysis of historical data shows that the association of silage as a source of contamination in ruminants should be re-evaluated. The most common clinical presentation was encephalitis (64.8% of the animals, 1839/2837), followed by abortion (21.3% of the animals, 604/2837). The mortality rate was high despite treatment. Overall, the mean morbidity, case fatality rate, and abortion rate were 12.6%, 50.6%, and 12.8%, respectively. Meta-analysis of the subgroups revealed a Hedges’ g value of −4.60 for the abortive form, indicating greater morbidity than mortality in this form. In contrast, the encephalitic form was characterized by a higher case fatality rate than morbidity (Hedges’ g 9.46). Literature gaps exist since most reported outbreaks are from the twentieth century and only from a few countries. There is a lack of information on the current prevalence, consequences, and effectiveness of antimicrobial treatment of listeriosis in domestic ruminants. There is also an incomplete picture of the prevalence of *Listeria* infection worldwide.

## Introduction

*L. monocytogenes* is the foodborne pathogen associated with the highest case-fatality in humans in the western hemisphere (Charlier et al. 2017; EFSA 2023). More than 40 animal species have been reported to be affected by *L. monocytogenes* (World Organisation for Animal Health 2021). Listeriosis occurs most commonly in humans and farmed ruminants (Dhama et al. 2015). *L. monocytogenes* is a cause of concern due to economic losses when livestock is affected and due to public health and food safety issues.

Ruminants play a key role in the epidemiology of *L. monocytogenes*, as *L. monocytogenes* Clonal Complex 1 (CC1) (a hypervirulent clone and the most associated with clinical cases in humans) spread globally from North America through cattle trade (Moura et al. 2021). Dairy ruminants harbor in their intestine *L. monocytogenes* hypervirulent clones (Palacios-Gorba et al. 2021), which are also overrepresented in dairy products (Maury et al. 2019). Subsequent studies suggested that hypervirulent *L. monocytogenes* clones overrepresentation in dairy products arises from contamination during or after milking at the farm in the context of intramammary infection and fecal or environmental contamination of the udder surface (Maury et al. 2019; Espí-Malillos et al. 2025, 2025a). In dairy ruminants, *L. monocytogenes* fecal shedding can lead to milk contamination and subsequent outbreaks such as those reported in studies (Linnan et al. 1988; Nüesch-Inderbinen et al. 2021). Fecal shedding of *L. monocytogenes* can also favor inter-host transmission, and contamination of agricultural environments and raw products (Schlech et al. 1983). These contaminated materials may then be introduced into food processing facilities, increasing the risk of foodborne listeriosis outbreaks. *L. monocytogenes* was first described as causing encephalitis in sheep in 1937 (Gill 1937). The first case of abortive listeriosis in cattle and ewes was reported in 1939 in the USA and 1940 in the UK, respectively (Graham 1938; Paterson 1940). The other pathogenic *Listeria* species, *L. ivanovii*, almost exclusively affects ruminants (Quereda et al. 2021). Ruminants have been implicated as a reservoir of hypervirulent strains (Palacios-Gorba et al. 2021, 2023). Rates of *L. monocytogenes* infection in human are low (three to six cases per million population per year (Scallan et al. 2011; EFSA 2023) in the general population of Europe and North America, and cases are concentrated among high-risk groups (older age, immunosuppression, and pregnant). Animal listeriosis cases manifest frequently as outbreaks. However, since listeriosis in animals is not a notifiable disease in many countries, it is unclear how common it is, what its clinical and pathological patterns are, and what its outcomes are. Clinical features of listeriosis in ruminants include encephalitis, myelitis, abortion, septicemia, and gastroenteritis. Localized forms are also reported in ruminants, such as mastitis, iritis, uveitis, and conjunctivitis in ruminants (Quereda et al. 2021; Barbuddhe et al. 2022). The role of feeding *Listeria*-contaminated silage has been historically reported as the primary infection source (Walland et al. 2015).

Given the relevance of *L. monocytogenes* for human and animal health, it is essential to understand the prevalence, geographic distribution, and genetic characteristics of the strains circulating in animals and their environment to map the reservoirs of the bacterium, identify sources of infection and improve surveillance. Some studies have examined the clinical features and outcomes of listeriosis in ruminants (Kumar et al. 2007; Bundrant et al. 2011; Fairley et al. 2012; Dreyer et al. 2015; Garcia et al. 2016; Matto et al. 2017). However, most of these were single-farm studies, examined diverse populations, and used different diagnostic criteria and treatments. A precise analysis of the disease in animals from heterogeneous timeframes and geographical areas comparing epidemiology, clinical and pathological parameters, antibiotic usage, outcome, and genetic characteristics of the strains involved is lacking. Thus, current therapeutic guidelines and conclusions about epidemiology and clinical presentation of listeriosis in domestic animals are not evidence-based, and a systematic review and meta-analysis using an evidence-based approach is needed. Compiling antibiotic treatment outcomes in domestic animals is necessary to provide clinicians with proven recommendations for antibiotic treatment.

Here, we conducted a systematic review and meta-analysis of listeriosis in ruminants to collect all the scientific data from published studies and summarize the estimates of epidemiologic, clinical, pathological, and genetic parameters, as well as antibiotic efficacy to support practitioners’ and policymakers’ efforts to prevent and control this zoonotic infection.

## Material and methods

### Protocols and registration

This systematic review was created in accordance with the Preferred Reporting System for Systematic Reviews and Meta-analyses (PRISMA) (Moher et al. 2009). The protocol has been registered at the International Prospective Register of Systematic Reviews (PROSPERO) with reference number CRD420251018482.

### Search strategy

Comprehensive, structured literature searches were conducted *via* the databases PubMed, Web of Science, and Scopus. The literature search was limited to English language studies with no publication date limits (until the end of December 2024). The electronic search was performed using the phrases lister* AND (infection OR case OR outbreak OR disease OR Clinical) AND (ruminant OR cow OR bovine OR cattle OR bull OR sheep OR ovine OR ewe OR goat OR caprine OR buck OR lamb OR buffalo) for Scopus and Web of Science. Search terms with Mesh terms: (lister*[MeSH Terms]) AND (infection [MeSH Terms] OR case*[MeSH Terms] OR outbreak[MeSH Terms] OR disease[MeSH Terms] OR clinical[MeSH Terms]) AND (ruminant OR cow OR bovine OR cattle OR bull OR sheep OR ovine OR ewe OR goat OR caprine OR buck OR lamb OR buffalo [MeSH Terms]) were searched in PubMed. Two authors (ILA and JJQ) independently reviewed the titles and abstracts of all studies and excluded those that did not meet the selection criteria. Discrepancies were resolved by discussion between the authors, until a consensus was reached.

### Study selection

First, a primary literature review was performed. Next, the abstracts and titles were examined to assess and discard articles that were not relevant to this study. After this, the remaining articles were downloaded as full-text articles for further review. Based on the inclusion and exclusion criteria, only studies meeting these criteria were included in this systematic review and meta-analysis.

### Inclusion and exclusion criteria

The basic inclusion criteria were that the studies had to report epidemiological, clinical, pathological, and analytical data and/or antibiotic treatment and outcomes in animals diagnosed with listeriosis. Observational analytical studies were included. Experimental studies were excluded. The following study types were also excluded from this review: reviews, letters, biographies, directories, editorials, lectures, commentaries, abstracts, and meta-analyses. Studies that did not focus on the subject of study, or used wild animals were also excluded. Table 1 shows all the study inclusion and exclusion criteria. The titles and abstracts of the articles identified from the search results were assessed in the context of the inclusion criteria. The articles included were then evaluated in relation to the exclusion criteria.

**Table 1.** Study inclusion and exclusion criteria.

Inclusion	Exclusion
Descriptive analytical studies	Experimental studies, reviews, letters, biographies, directories, editorials, lectures, commentaries, abstracts, meta-analysis
*Listeria* spp. infection (neurolisteriosis, maternal-neonatal infections, gastroenteritis, and septicemia cases)	Comorbidities/coinfection
Articles must include epidemiological, clinical, or pathological features, laboratory data, or antibiotic treatment and outcomes	Localized infection cases
All years included (up to December 2024)	No epidemiological, clinical, pathological, or analytical information or antibiotic treatment data
Articles in English	Wild animal listeriosis
	No clinical data was presented (only anatomopathological data was presented)

### Clinical presentation

Studies describing gastroenteritis, septicemic, myelitis, central nervous system, fetal-placental listeriosis were included. Studies dealing with local infections such as lymphadenitis, mastitis, or eye infections due to *Listeria* spp. were discarded.

### Data extraction

Tables 2, 3, 4 and 5 were used to organize the information gained from each study. Table 2 contains information regarding publication year, country, type of study, affected species, silage use, and season. Table 3 displays data from clinical and pathological characteristics. Table 4 contains information on morbidity, case fatality rate, and abortion rate. Table 5 contains information on antibiotic treatment and outcome as well as strain typification. Articles were reviewed by authors ILA and JJQ. Disputes were resolved by AGM and JG. ILA and JJQ collected the necessary data from the chosen articles for subsequent evaluation, and AGM and JG cross-checked data for suitability.

**Table 2.** Information regarding publication year, country, type of study, affected species, silage use, and season of the included studies.

Study	Year	Country	Type study	Species	Silage	Season
Graham	1938	USA	Case report	Sheep and cattle	No information	na
Biester	1939	USA	Case report	Sheep	No information	Spring
Paterson	1940	United Kingdom	Case report	Sheep	No information	Winter
Harbour	1941	United Kingdom	Case report	Cattle	No information	na
Cole	1946	USA	Case report	Cattle	No information	Winter
Boucher	1946	USA	Case report	Cattle	No information	Winter
Thorp	1947	USA	Case report	Cattle	No information	Spring
Pounaen	1947	USA	Case report	Cattle	No information	Autumn
Viswanathan	1950	India	Compiled cases	Sheep	No information	na
Ferguson	1951	USA	Case report	Cattle	No information	Winter
Zink	1951	USA	Case report	Cattle	No information	Summer/autumn/winter
Clapp	1953	Australia	Case report	Sheep	No information	Winter
Eveleth	1953	USA	Case report	Sheep	No tested for *L. monocytogenes*	Winter/spring
Stockton	1954	USA	Case report	Cattle	No information	na
Smith	1955	USA	Case report	Cattle	No information	Spring
Diplock	1957	Australia	Case report	Sheep	No information	Winter
Young	1958	USA	Case report	Cattle	No tested for *L. monocytogenes*	Winter
Osebold	1960	USA	Case report	Cattle	No information	Winter and summer
Gitter	1965	United Kingdom	Case report	Sheep	1 farm: no tested for *L. monocytogenes*. 2 out 3 farms did not have access to silage	Winter
Gates	1967	USA	Case report	Sheep	No information	Spring
McDonald	1967	Australia	Case report	Sheep	No information	Autumn
Wood	1972	Canada	Case report	Goat	No information	Spring
Oshima	1974	Japan	Case report	Cattle	No information	Spring
Macleod	1974	United Kingdom	Case report	Sheep	No information	Winter
Dennis	1975	Australia	Compiled cases	Sheep	No information	Winter/spring
du Toit	1977	South Africa	Case report	Goat	No information	Spring
Groonstool	1979	Norway	Case report	Sheep	Contaminated with *L. monocytogenes*	Autumn/winter/spring
Vandegraaff	1981	Australia	Case report	Sheep	No information	Autumn/winter/spring
Price	1981	USA	Case report	Cattle	No tested for *L. monocytogenes*	Winter/spring
Loken	1982	Norway	Case report	Goat	No contaminated with *L. monocytogenes*	Autumn
Wardrope	1983	United Kingdom	Case report	Sheep	No contaminated with *L. monocytogenes*	Winter
Yousif	1984	Iraq	Case report	Sheep and goat	No information	na
Meredith	1984	South Africa	Case report	Sheep	No information	Winter/spring
Low	1985	United Kingdom	Case report	Sheep	Contaminated with *L. monocytogenes*	Winter
West	1987	United Kingdom	Case report	Cattle	No tested for *L. monocytogenes*	Winter
Reuter	1989	Australia	Case report	Sheep	No tested for *L. monocytogenes* (silage in 1 out of 2 outbreaks)	Summer
Seaman	1990	Australia	Case report	Sheep	No information	Spring
Sergeant	1991	Australia	Case report	Sheep	No tested for *L. monocytogenes*	Winter
Vazquez-Bolana	1992	Spain	Case report	Sheep	Contaminated with *L. monocytogenes* (1.9 × 10^6 CFU/g)	Winter
Akpavie	1992	Nigeria	Case report	Cattle	No information	na
Scott	1993	United Kingdom	Prospective cohort	Sheep	No tested for *L. monocytogenes*	Winter
Green	1994	United Kingdom	Prospective cohort	Sheep	No contaminated with *L. monocytogenes*	Winter
Nash	1995	USA	Case report	Sheep	Contaminated with *L. monocytogenes*	Winter/spring
Chand	1999	India	Case report	Sheep	No information	Winter
Ayars	1999	USA	Case report	Cattle	Contaminated with *L. monocytogenes*	na
al-Dughaym	2001	Saudi Arabia	Case report	Sheep	No information	Autumn/winter
Braun	2002	Switzerland	Compiled cases	Sheep and goat	No information	na
Clark	2004	New Zealand	Compiled cases	Sheep	*L. monocytogenes* in 85% cases	Winter
Otter	2004	United Kingdom	Compiled cases	Sheep	No tested for *L. monocytogenes*	Winter
Wagner	2005	Austria	Case report	Sheep	Contaminated with *L. monocytogenes* (10^5 CFU/g)	Winter/spring/summer
Sahin	2006	Turkey	Case report	Sheep	No information	Winter/spring
Schweizer	2006	Switzerland	Compiled cases	Cattle	No information	Spring
Kumar	2006	India	Case report	Sheep	No silage feeding	Winter
Bunarant	2011	USA	Case report	Cattle	No contaminated with *L. monocytogenes*	na
Fairley	2013	New Zealand	Case report	Cattle	No tested for *L. monocytogenes*	Winter
Dreyer	2015	Switzerland	Case report	Sheep	No contaminated with *L. monocytogenes*	Winter
Garcia	2016	Argentina	Case report	Cattle	Contaminated with *L. monocytogenes*	na
Matto	2017	Uruguay	Case report	Cattle	No contaminated with *L. monocytogenes*	na
Prado	2019	Brazil	Case report	Buffalo	No information	Autumn/winter
Whitman	2020	USA	Case report	Cattle	Contaminated with *L. monocytogenes*	Winter/spring
Osman	2021	Saudi Arabia	Case report	Sheep	No information	Winter
Ribeiro	2022	Brazil	Case report	Sheep	Contaminated with *L. monocytogenes*	Spring
Ali	2024	Sultanate of Oman	Case report	Sheep and goat	No information	Summer/autumn

‘na’ means information not available in the publication.

**Table 3.** Information regarding clinical and pathological characteristics of the included studies.

Study	Clinical syndromes	Age	Body temperature	Blood findings	Cerebrospinal fluid	Histological findings	Gross pathological lesions
Graham 1938	Encephalitis	na	na	na	na	Meningoencephalitis	Cloudy cerebrospinal fluid with congestion of the meninges, ophthalmia
Biester 1939	Encephalitis	Lambs	High	na	na	Encephalitis	No gross lesions, grayish cornea
Paterson 1940	Abortion	na	na	na	na	na	No gross lesions
Harbour 1941	Diarrhea and septicemia	2 days old	na	na	na	Hepatitis, abomasitis (brain was not examined due to the absence of neurological symptoms)	Congestion, hemorrhages and ulcers in the abomasum, congestion of the ileum, liver with necrotic foci
Cole 1946	Encephalitis	7 years old	High	No leucocytosis	High leucocyte counts	Meningoencephalitis	Congestion of brain and meninges, cornea dried and eroded
Boucher 1946	Encephalitis	10 years old	Normal	na	na	na	No gross lesions
Thorp 1947	Encephalitis	na	na	na	na	Encephalitis	Excess cerebrospinal fluid, hemorrages under epicardium, congestion mucous membrane abomasum and small intestine
Pounden 1947	Encephalitis	3–4 months old	High (9/27)	na	na	na	Hemorrages in heart and kidneys, hyperemia and edema lungs, meningitis, conjunctivitis
Viswanathan 1950	Encephalitis	na	High	na	na	na	Congestion of meninges
Ferguson 1951	Abortion	na (fetus of 7 months old)	na	na	na	na	na
Zink 1951	Encephalitis	na	High (1/6)	na	na	na	Congestion of meninges, excess of cerebrospinal fluid, cerebrum congested ana edematous
Eveleth 1953	Abortion	na	na	na	na	na	na
Clapp 1953	Encephalitis	na	High	na	na	na	No gross lesions
Stockton 1954	Abortion	9 years old	na	na	na	na	na
Smith 1955	Encephalitis and abortion	3 years old	High	na	na	Aborted cow: endometritis, no CNS changes	No gross lesions in the aborted cow; Fetuses: reddened, gelatinous infiltration of muscles and organs, pleural and peritoneal fluids with yellow flocculent material
Diplock 1957	Abortion	> 2 years old (abortion 3 weeks before lambling)	na	na	na	na	No gross lessions in aborted foetuses
Young 1958	Abortion and diarrhea	3–10 years old (aborted animals were young 3–4 years)	na	Leucocytosis (1/16), monocytosis (5/16), leukopenia (5/16)	na	na	Edema in mesentery
Osebold 1960	Abortion	3–9 years old	High	na	na	na	Fetuses with red watery fluid in cavities
Gitter 1965	Encephalitis-septicemia	Lambs: started dying to 2–7 days old	na	na	na	Lambs: septicemia: necrotic foci in liver and spleen. Suppurative lymphadenitis; ewes: meningoencephalitis	Ewes: no gross lesions; lambs: white miliary lesions in the liver; lungs, spleen and kidneys congested
Gates 1967	Myelitis	3–6 months old	Normal	na	na	Meningitis at cervical, thoracic or lumbar level	No gross lesions
McDonald 1967	Abortion	na (abortion 1–4 weeks before lambling)	na	na	na	na	Fetuses: necrosis liver (25/31), subcutaneous edema (23/28), excess body fluid (22/28), blood clots brain (15/30)
Wood 1972	Encephalitis	na	High (1/3)	Leucocytosis (2/3), neutrophilia (3/3), monocytosis (2/3)	na	Meningoencephalitis	Focal grey discolouration and malacia of the brain stem in 2/3 goats
Macleod 1974	Abortion	na (abortion 1–7 weeks before lambling)	na	na	na	Placentitis	Fetus: autolyses, sanguineus fluid in abdominal cavity. Cotyledons reddened and necrotic
Oshima 1974	Encephalitis	8 years old	Normal	No leucocytosis	na	Encephalitis and inflamation of cornea, iris and sclera	Eye hemorrhage
Dennis 1975	Abortion	na (abortion 2 weeks before lambling)	na	na	na	Hepatitis, abomasitis	Fetuses: autolysis (20/40), subcutaneous oedema, hydrothorax and hydroperitoneum, distended abdomen, enlarged livers with small necrotic foci, small abomasal erosions, enlarged mesenteric lymph nodes (30/40)
du Toit 1977	Encephalitis	na	High	na	na	Meningoencephalitis, myocarditis	Congestion of meninges, oedema of the brain
Groonstool 1979	Encephalitis-abortion	Encephalitic outbreak (ewes 2–9 years, hoggs 8 months old), abortion outbreak (1–11 year) (abortion days before lambling)	na	na	na	na	na
Vandegraaff 1981	Encephalitis	All ages affected (highest incidence was observed in lactating ewes and weaners)	na	na	na	Meningoencephalitis	Most without gross abnormalities. Some: yellowing of the meninges
Price 1981	Diarrhea and septicemia	na	High (2/2)	na	na	Signs of septicemia, no lesions in the brain	na
Loken 1982	Encephalitis-septicemia	na	High	na	na	Encephalitis, hepatitis, nephritis	No gross lesions
Wardrope 1983	Encephalitis	5 weeks old	Normal	Leucocytosis (2/9)	na	Meningoencephalitis	No gross lesions
Yousif 1984	Encephalitis	na	Normal	na	Only for culture	Meningoencephalitis, necrotic foci in liver	No gross lesions apart from conjunctivitis
Meredith 1984	Encephalitis	na	na	na	na	Meningoencephalitis	1/3 sheep had thickened meninges
Low 1985	Encephalitis, abortion, diarrhea and septicemia	Adult ewes	High	Neutrophilia (2/3), hypocalcemia (5/5)	na	Meningoencephalitis, ulcerative colitis	No gross lesions in the CNS or aborted material. Mucoid enteritis, hemorrhages on heart and spleen
West 1987	Encephalitis	2 years old	Normal	na	na	Meningoencephalitis	No gross lesions apart from keratitis
Reuter 1989	Encephalitis	Outbreak 1: different ages; Outbreak 2: 15–19 months old	na	na	na	Meningoencephalitis	No gross lesions
Seaman 1990	Myelitis	From 3 months to 6 years old	na	na	na	Myelitis (inflammatory lesions in the brain in 3/6)	Excess of clear cerebrospinal fluid was noted in the cranial cavity (3/6)
Sergeant 1991	Abortion	5 years old	na	na	na	Lamb (1/1): hepatitis	Lamb (4/9): hepatitis necrotic foci, hydrothorax, ascites
Vazquez-Boland 1992	Encephalitis	Mostly reproductive pregnant ewes in the last third of pregnancy	na	na	na	na	na
Akpavie 1992	Encephalitis and abortion	na	High	na	na	Meningitis	No gross lessions
Scott 1993	Encephalitis	2.5–96 months old	na	na	Leucocytosis (17/21), high protein concentration (18/21)	Meningoencephalitis	na
Green 1994	Encephalitis	6–12 weeks old	na	na	na	na	na
Nash 1995	Encephalitis and septicemia	2–12 years old	na	na	na	Encephalitis	na
Chand 1999	Abortion	na	na	na	na	na	Lamb: autolyses, clear-sanguineous fluid in cavities (12/19), hepatitis necrotic foci (7/19), erosions abomasal mucose (4/19), enlarged mesenteric lymph nodes (13/19)
Ayars 1999	Encephalitis	19–32 months old	High (3/5)	na	na	Encephalitis	No gross lesions
al-Dughaym 2001	Encephalitis	na	High	na	na	Meningoencephalitis, lymphadenitis, spleen congested with marked haemosiderosis and depletion of white pulp, liver vascular degeneration and lungs congested with areas of alveolar haemorrhage, keratoconjunctivitis	Congestion and turbid meningeal oedema, lungs with multiple areas of haemorrhage, keratoconjunctivitis
Braun 2002	Encephalitis	From 8 weeks to 7 years old	High 21/67, low 2/67	Leucocytosis (5/62) high hematocrit (16/61), high total protein (33/61), high bilirubin (39/61), high urea (28/63), metabolic acidosis (28/53)	Leucocytosis (8/9), high protein concentration (9/9)	Meningoencephalitis	na (detailed necropsy is not reported, keratoconjunctivitis reported)
Clark 2004	Diarrhea	Different age	na	na	na	Abomasitis, enteritis	Reddening and sometimes haemorrhage, ulceration or erosion in the abomasal mucosa. The intestine also often showed reddening
Otter 2004	Diarrhea	Pregnant ewes and 9 months old ewes	na	na	na	Abomasitis (6/6) and typhlocolitis (3/6)	Reddening and ulceration in the abomasal mucosa. Ulcerative fibrinopurulent typhlocolitis
Wagner 2005	Encephalitis, abortion and septicemia	Pregnant ewes (Abortion 10–14 days before lambing)	na	na	na	Meningoencephalitis, pneumonia, enteritis, hepatitis, nephritis and placentitis. Fetus: hepatitis	na
Sahin 2006	Abortion	na	na	na	na	Hepatitis, focal necrosis lungs	White necrotic foci on the liver, clear to sanguineous fluid in the body cavities
Schweizer 2006	Encephalitis	From 6 months to 10 years old	High 44/94, low 14/94	Leucocytosis (41/94) high hematocrit (24/94), high total protein (54/94), high bilirubin (6/94), high urea (26/94), high AST (60/91) High CGT (60/91) metabolic acidosis (31/94)	Leucocytosis (57/74), high protein concentration (51/74)	na	na (detailed necropsy is not reported, keratoconjunctivitis reported)
Kumar 2006	Encephalitis	Different age	High in few cases	na	Total proteins elevated	Meningoencephalitis. In some animals hepatitis	Mild cerebral congestion, necrotic foci were observed in liver in one case only, corneal opacity
Bundrant 2011	Encephalitis	na	na	na	na	Encephalitis	na
Fairley 2013	Diarrhea	10–11 months old	na	na	na	Abomasitis, enteritis, and mesenteric lymphadenitis	Abomasal mucosa red
Dreyer 2015	Encephalitis-septicemia	3 days old (lambs), ewes no available	na	na	na	Ewes: encephalitis. Lamb: pyogranulomas in organs	Lamb: Pyogranulomas were found macroscopically and/or histopathologically in multiple organs (lungs, liver, spleen, kidney and lymph nodes)
Garcia 2016	Diarrhea	1 year old	High	na	na	Enteritis, lymphadenitis and hepatitis	1 L of translucent yellow fluid in the abdominal cavity and severe congestion of the entire digestive tract
Matto 2017	Encephalitis	2 years old	na	na	na	Meningoencephalitis	na
Prado 2019	Encephalitis	<40 days	na	na	na	Meningoencephalitis	No gross lessions
Whitman 2020	Abortion	Heifers (Abortion in the 3rd trimester)	na	na	na	Fetus: necrotic foci liver	na
Osman 2021	Encephalitis and abortion	Different age	Fever only at the early stages of the disease	na	na	na	na
Ribeiro 2022	Encephalitis	5–6 months old	na	na	na	Encephalitis	No gross lessions
Ali 2024	Encephalitis	From less than 6 months to 2–3 years	High	na	na	Microabscesses and perivascular cuffs in the pons, medulla oblongata and anterior spinal cord	In most cases, no significant gross lesions were observed except slight meningeal congestion of the brain stem.

‘na’ means information not available in the publication.

**Table 4.** Information regarding morbidity, case fatality rate, and abortion rate.

Study	Morbidity	Case fatality rate	Abortion rate
Graham 1938	Outbreak 1: aprox 12% (30/250); outbreak 2: aprox 6% (6/100)	na (assumed aprox 100%)	na
Biester 1939	11.7% (258/2200)	98.8% (255/258)	na
Paterson 1940	50% (16/32)	Abortive	50% (16/32)
Harbour 1941	na	na	na
Cole 1946	na	100% (1/1)	na
Boucher 1946	6.6% (1/15)	100% (1/1)	na
Thorp 1947	na	100% (3/3)	na
Pounden 1947	100% (27/27)	29.6% (8/27)	na
Viswanathan 1950	Outbreak 1: 5.8% (35/595); outbreak 2: 14.7% (119/805) ; outbreak 3: 6.2% (56/890); outbreak 4: 10% (20/200)	Outbreak 1: 97.1% (34/35); outbreak 2: 98.3 (117/119) ; outbreak 3: 96.4% (54/56); outbreak 4: 90% (18/20)	na
Ferguson 1951	6% (3/50)	Abortive	6% (3/50)
Zink 1951	27% (6/22)	33.3% (2/6)	na
Eveleth 1953	5.8% (64/1100)	Abortive	5.8% (64/1100)
Clapp 1953	Outbreak 1: 0.8% (8/1000); outbreak 2: 0.3% (1/300); outbreak 3: 0.4% (3/750)	na	na
Stockton 1954	9% (1/11)	Abortive	9% (1/11)
Smith 1955	na	Abortive	No information
Diplock 1957	16% (30/180)	Abortive	16% (30/180)
Young 1958	8.4% (21/250)	none	8.4% (21/250)
Osebold 1960	Herd A: 2% (7/400); herdB: 7.7% (10/130), 31% (40/130), 6.15% (8/130); herdC: 4%(11/300), 6% (17/300), 5% (15/300), 0.09% (3/300)	Abortive	Herd A: 2% (7/400); herdB: 7.7% (10/130), 31% (40/130), 6.15% (8/130); herdC: 4%(11/300), 6% (17/300), 5% (15/300), 1% (3/300)
Gitter 1965	Lambs: 16.7% (60/360); ewes 1% (3/280)	100%: (60/60 and 3/3)	na
Gates 1967	2.8% (4/145)	na	na
McDonald 1967	16.8% (37/220)	Abortive	16.8% (37/220)
Wood 1972	18.7% (3/16)	100% (3/3)	na
Macleod 1974	18% (18/100)	na	18% (18/100)
Oshima 1974	na	100% (1/1)	na
Dennis 1975	5–22%	Abortive	5–22%
du Toit 1977	na	11.4% (8/70)	na
Groonstool 1979	Encephalitic outbreak:12.2% (13/106); abortion outbreak: 33.3% (16/48)	Encephalitic outbreak: 46.1% (6/13); abortion outbreak: 2% (1/48)	33.3%(16/48)
Vandegraaff 1981	0.2–8%	Approx 100%	na
Price 1981	33% (66/200)	12% (8/66)	na
Loken 1982	Encephalitic outbreak: 14% (7/50); septicemic outbreak: 34% (17/50)	Encephalitic outbreak: 14.2% (1/7); septicemic outbreak: 5.9% (1/17)	na
Wardrope 1983	2% (9/440)	na	na
Yousif 1984	Sheep: 6.7% (105/630); goats: 30% (51/170)	Sheep: 89.5% (94/105); goats: 70.6% (36/51)	na
Meredith 1984	na	3.6% (10/275)	na
Low 1985	na	na	No information
West 1987	1.4% (1/70)	100% (1/1)	na
Reuter 1989	Outbreak 1: 1.9% (14/750); outbreak 2: 9.3% (40/430)	na	na
Seaman 1990	1.2% (37/3000)	0.8–2.5%.	na
Sergeant 1991	na	na	12%
Vazquez-Boland 1992	11.8% (53/450)	94.3% (50/53)	na
Akpavie 1992	9.9% (39/391)	na	No information
Scott 1993	<1%	76.2% (16/21)	na
Green 1994	0.5% (21/4413)	85.7% (18/21)	na
Nash 1995	na	Ewes: 3.1% (29/936); lambs: 1.3% (17/ 1262).	na
Chand 1999	na	na	9.4% (24/254)
Ayars 1999	1% (5/478)	20% (1/5)	na
al-Dughaym 2001	7.1% (149/2100)	33% (50/149)	na
Braun 2002	na	Treated animals: 74%; all animals, treated and non treated: 85%	na
Clark 2004	na	na (Mortality rates ranged from 0.16% to 3.3%)	na
Otter 2004	6 flocks: 4.4% (11/250), 5.7% (12/211), 13.2% (18/136), 20% (40/200), 1.7% (4/230), 1.3% (4/300)	6 flocks: 100% (11/11), 41.6% (5/12), 33.3% (6/18), 35% (14/40), 25% (1/4), 100% (4/4)	na
Wagner 2005	25% (14/55)	35.7% (5/14)	25% (14/55)
Sahin 2006	10% (12/120)	na	10%(12/120)
Schweizer 2006	na	29%	na
Kumar 2006	7.89% (69/875)	89.85% (62/69)	na
Bundrant 2011	2.8% (9/315)	78% (7/9)	na
Fairley 2013	2.1% (3/140)	33% (1/3)	na
Dreyer 2015	na	na	na
Garcia 2016	20% (40/200)	32.5% (13/40)	na
Matto 2017	1.5% (1/64)	100% (1/1)	na
Prado 2019	6.38%(3/47)	100%(3/3)	na
Whitman 2020	3% (28/936)	none	3% (28/936)
Osman 2021	Encephalitic outbreak: 57.14% (400/700); abortive outbrak: 6.25% (5/80)	87.5% (350/400)	6.25% (5/80)
Ribeiro 2022	0.79% (7/878)	71.42% (5/7)	na
Ali 2024	8 outbreaks: 5.3% (3/56), 7.8% (6/77), 9.5% (4/42), 2.9% (4/140), 20% (6/30), 5% (2/40), 2.9% (2/70), 37.5% (3/8)	8 outbreaks: 100% (3/3), 66.7% (4/6), 25% (1/4), 100% (4/4), 100% (6/6), 50% (1/2), 100% (2/2), 100% (3/3)	na

‘na’ means information not available in the publication.

**Table 5.** Information regarding antibiotic treatment and outcome and strain typification of the included studies.

Study	Clinical syndromes	Treatment and efficacy	Genetic characteristics
Graham 1938	Encephalitis	none	*Listerella*
Biester 1939	Encephalitis	none	*Listerella*
Paterson 1940	Abortion	none	*Listerella* (based on hemolysis zone in blood agar, it should be *L. ivanovii)*
Harbour 1941	Diarrhea and septicemia	none	*Listerella*
Cole 1946	Encephalitis	none	*Listerella monocytogenes*
Boucher 1946	Encephalitis	none	*Listerella monocytogenes*
Thorp 1947	Encephalitis	none	*Listerella monocytogenes*
Pounaen 1947	Encephalitis	Sulfathiazole and sulfanilamide in n = 1 / the treated animal died 4 h later	*Listerella monocytogenes*
Viswanathan 1950	Encephalitis	none	*Listerella monocytogenes*
Ferguson 1951	Abortion	none	*L. monocytogenes*
Zink 1951	Encephalitis	Sulfanilamide and penicillin in n = 3/ 2 died; 1 animal aureomycin	*L. monocytogenes*
Clapp 1953	Encephalitis	none	*L. monocytogenes*
Eveleth 1953	Abortion	none	*L. monocytogenes*
Stockton 1954	Abortion	none	*L. monocytogenes*
Smith 1955	Encephalitis and abortion	Penicillin / cow was finally killed	*L. monocytogenes*
Diplock 1957	Abortion	none	*L. monocytogenes*
Young 1958	Abortion and diarrhea	Sulfathiazole, sulfanilamide, tetracycline, streptomycine / no more clinical signs observed	*L. monocytogenes*
Osebold 1960	Abortion	none	*L. monocytogenes*
Gitter 1965	Encephalitis-septicemia	New born lambs: intramuscular penicillin and oxytetracycline; lambs under 1 week age: intramuscullar penicillin and cloramphenicol / all died (60/60)	*L. monocytogenes*
Gates 1967	Myelitis	none	*L. monocytogenes*
McDonald 1967	Abortion	none	*L. monocytogenes*
Wood 1972	Encephalitis	Intravenously (chloranpheenicol, oxytetracycline), penicillin-streptomicyn no specified/ 3/3 treated died	*L. monocytogenes*
Oshima 1974	Encephalitis	none	na
Macleod 1974	Abortion	Penicillin/favourable response (no numbers given)	*L. monocytogenes* serotype 5 (*L. ivanovii*)
Dennis 1975	Abortion	none	*L. monocytogenes* serotype 5 (*L. ivanovii*)
du Toit 1977	Encephalitis	none	*L. monocytogenes*
Groonstool 1979	Encephalitis-abortion	none	*L. monocytogenes* (encephalitic form (serotypes 1 and 4), abortive (serotype 1))
Vandegraaff 1981	Encephalitis	none	*L. monocytogenes*
Price 1981	Diarrhea and septicemia	Vaccination (bacterin) + ampicillin / no new cases appeared after treatment	*L. monocytogenes*
Loken 1982	Encephalitis-septicemia	Penicillin (septicemia goats), peniccilin-streptomycin (CNS goats)/ 10/10 treated and survived (septicemia), 6/7 treated and survived (CNS)	*L. monocytogenes* (serotype 4)
Wardrope 1983	Encephalitis	none	*L. monocytogenes* (serotype 1/2)
Yousif 1984	Encephalitis	80 animals treated with oxytetracycline / no numbers given	*L. monocytogenes*
Meredith 1984	Encephalitis	Chloramphenicol/ All died (10/10)	*L. monocytogenes*
Low 1985	Encephalitis, abortion, diarrhea and septicemia	Oxytetracycline, penicillin /no numbers given	*L. monocytogenes* (serotype 1/2)
West 1987	Encephalitis	Penicillin, chlortetracycline / no effect	*L. monocytogenes*
Reuter 1989	Encephalitis	none	*L. monocytogenes*
Seaman 1990	Myelitis	Penicillin/streptomycin/ no numbers given	*L. monocytogenes*
Sergeant 1991	Abortion	none	*L. ivanovii*
Akpavie 1992	Encephalitis and abortion	Cotrimoxazole / No numbers given: deaths and abortions stopped after treatment	*L. monocytogenes* (serotype 4b)
Vazquez-Bolana 1992	Encephalitis	Tetracycline, penicilin, gentamicin and spiramicin / 3/53 survived	*L. monocytogenes* (serotype 4b)
Scott 1993	Encephalitis	Dexamethasone + penicillin / (5/21) of listerial meningo-encephalitis responded to the high dose penicillin G treatment regimen	na
Green 1994	Encephalitis	Oxytetracycline + dexamethasone / 3/8 survived	*L. monocytogenes* (serotype 1/2b)
Nash 1995	Encephalitis and septicemia	none	*L. monocytogenes*
Chand 1999	Abortion	none	*L. ivanovii*
Ayars 1999	Encephalitis	Oxytetracycline, dexamethasone / 4/5 recovered	*L. monocytogenes* (it could not be isolated from the brain of the only dead bull)
al-Dughaym 2001	Encephalitis	Oxytetracycline / total recovered (99/99)	*L. monocytogenes*
Braun 2002	Encephalitis	36 animals received antibiotics and flunixin meglumine (cloramphenicol or oxytetracycline or gentamicin/ampicillin or penicillin) / Cured chloramphenicol (1/15), oxytetracycline (2/11), gentamicin-ampicillin (6/9), penicillin (1/1)	na
Clark 2004	Diarrhea	none	*L. monocytogenes*
Otter 2004	Diarrhea	none	*L. monocytogenes*
Wagner 2005	Encephalitis, abortion and septicemia	Penicillin + streptomycin / No numbers given	*L. monocytogenes*
Schweizer 2006	Encephalitis	87/94 treated with various antibiotics (penicillin G, oxytetracycline, amoxicillin, and amoxicillin and gentamicin combined) / Cured oxytetracycline (16/21), chloranphenicol (10/18), Penicillin G (6/10), Amoxicillin (4/5), florfenicol (1/1), gentamicin + amoxi + flunixin (25/32)	na
Sahin 2006	Abortion	none	*L. ivanovii* subsp. *ivanovii*.
Kumar 2006	Encephalitis	none	*L. monocytogenes*
Bundrant 2011	Encephalitis	4 received florfenicol 5 received ampicillin / (2/4 died and 5/5 died)	*L. monocytogenes*, Lineage III (serotype 4b)
Fairley 2013	Diarrhea	Oxytetracycline/ 2/2 recovered	*L. monocytogenes*
Dreyer 2015	Encephalitis-septicemia	Antibiotic treatment (no specifications)	*L. monocytogenes*, CC4, Linage I (serotype 4b)
Garcia 2016	Diarrhea	none	*L. monocytogenes* (serotype 1/2c from the gallbladder and serotype 1/2b from the spoiled silage)
Matto 2017	Encephalitis	none	*L. monocytogenes* (serotype 4b)
Prado 2019	Encephalitis	penicillin/ no effect	*L. monocytogenes*
Whitman 2020	Abortion	none	*L. monocytogenes* (3 different strains, 2 Lineage I and 1 Lineage III)
Osman 2021	Encephalitis and abortion	Penicillin + dihydrostreptomycin sulphate and flunixin meglumine / no numberrs given	*L. monocytogenes*
Ribeiro 2022	Encephalitis	Oxytetracycline / 2/2 recovered	*L. monocytogenes*
Ali 2024	Encephalitis	none	*L. monocytogenes*

### Quality assessment measures

Quality assessment of case reports (Table S1a), cohort studies (Table S1b), and cases series (Table S1c) articles was carried out in accordance with The Joanna Briggs Institute critical appraisal tool (carried out by ILA and JJQ). Disputes were resolved by AGM and JG.

### Outcome measure

The primary outcome of the review is the epidemiological, clinical, pathological, and analytical manifestations of listeriosis in ruminants. The secondary outcome is the efficacy of the antibiotic treatment of listeriosis in ruminants.

### Descriptive statistics

Descriptive statistical analysis calculating the mean and 95% CI values of the proportion of morbidity and case fatality attributable to abortive, CNS, diarrheal, and septicemic manifestations of the disease was carried out. Furthermore, we carried out an analysis of the distribution of proportions of abortions among studies reporting on the abortive infection type.

### Meta-analysis

The Meta-Essentials tool was used to carry out all the meta-analysis calculations (Suurmond et al. 2017). Briefly, the tool requires the use of a set of Microsoft Excel workbooks that, following data input, automatically carry out the required statistics and generate the necessary tables and figures. Four clinical subgroups were established, relating to the clinical characteristics of the infection. These were abortive, central nervous system, diarrhoea, and septicemia. Data analysis was limited to studies in which the animals manifested a single clinical form of the disease (as such, very few included studies present data documenting the simultaneous manifestation of multiple clinical forms of the disease). The variables used for meta-analysis calculations were the proportion of morbidity and case fatality for each study, the pooled standard deviation for each clinical subgroup, and the total number of animals per group in the morbidity and case fatality groups.

The Hedges’ g statistic (the bias-adjusted standardized mean difference between morbidity and case fatality rate for each study) was calculated and presented *via* a Forest plot, using a random-effects model and a 95% confidence level. Hedges’ g values below 1 are summary indicators that morbidity > case fatality (in favor of morbidity), whilst values above 1 indicate that case fatality > morbidity (in favor of mortality) across the included studies. A value of 0 indicates that there was no difference between the proportion of morbidity and case fatality amongst the tested studies.

The data used to construct the Forest plot was also used to estimate the extent of heterogeneity *via* the calculation of the I^2^ value. As part of the subgroup analysis, the Tau statistic was determined, from which the prediction interval was calculated, permitting a description of the range of observed effect sizes about the mean.

An analysis of possible publication bias was carried out and displayed in the form of a funnel plot. The Meta-Essentials tool allows the calculation and adjustment for the estimate of the combined effect size in order to correct for potential publication bias. A detailed explanation of the calculation can be found in the Meta-Essentials user manual (van Rhee et al. 2015).

## Results and discussion

### Study selection process and results

**Figure 1.**
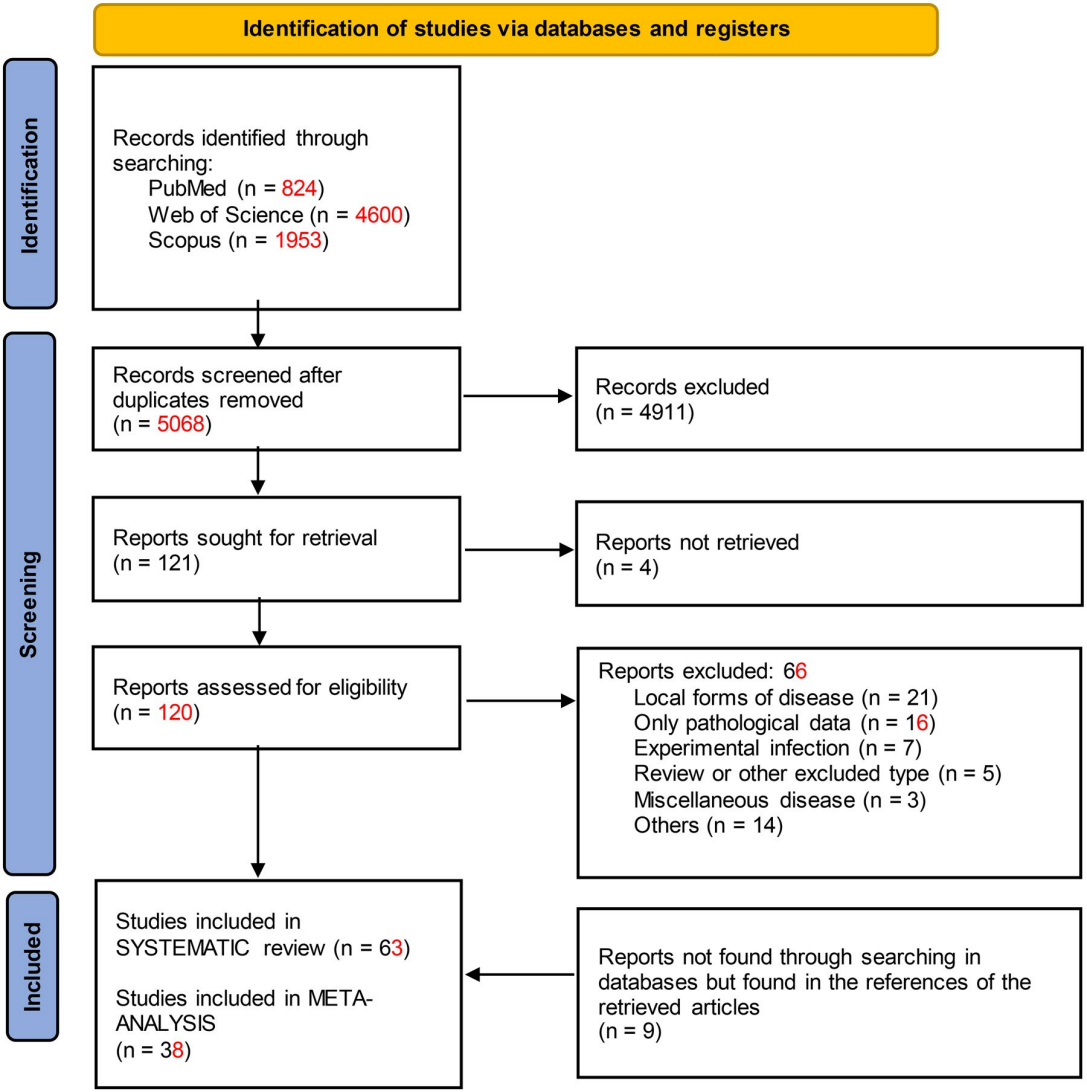
Flowchart of search strategy and study selection procedure.

Figure 1 shows the literature search results and selection process. We screened 5068 articles by reading the titles and abstracts and assessed 120 articles in full-text form. We determined that 63 and 38 publications met the systematic review and meta-analysis eligibility criteria, respectively (Figure 1).

### Study characteristics: type, country and year

Table 2 summarizes the characteristics of the 63 studies. There were 55 case report studies, six compiled case studies, and two prospective cohort studies. Table S1a shows the results of the critical appraisal tool of the included case reports. Table S1b shows the analysis of the included prospective studies. Table S1c shows the analysis of the included compiled clinical cases.

Nineteen studies were conducted in the USA, ten in the United Kingdom, eight in Australia, three in India and Switzerland, two in New Zealand, South Africa, Norway, Brazil and Saudi Arabia, and one in Japan, Argentina, Uruguay, Canada, Nigeria, Iraq, Turkey, Spain, Austria, and the Sultanate of Oman. Thus, data on outcomes of listeriosis in ruminants did not have worldwide coverage, despite the fact that some countries, such as India, have conducted relevant studies to better understand the ecology and epidemiology of the disease (Barbuddhe et al. 2022).

Importantly, current information on ruminant listeriosis is only represented by reports from research centers and does not include those in which diagnoses are made by practitioners, diagnostic laboratories, or health inspectors, for example, during antemortem examinations at slaughterhouses. Studies alerting about this situation were published in 1967 (Schwartz 1967) and 1986 (Wilesmith and Gitter 1986). Importantly, these studies described the incidence of listeriosis in Pennsylvania and Great Britain, respectively. The number of listeriosis cases is probably higher than shown in these reports since listeriosis cases were almost exclusively reported in the vicinity of Veterinary Colleges and Veterinary Experimental Stations (Schwartz 1967). The fact that animal listeriosis is not a reportable illness exacerbates the potential for underestimation. Furthermore, it is likely that cases may go unnoticed or be misdiagnosed in local laboratories due to a lack of diagnostic capabilities or a lack of knowledge about the condition.

Our data shows that 71.4% (45/63) of studies were conducted before the year 2000, and 88.9% (56/63) prior to 2016. Before 2016, the standard typification techniques for *L. monocytogenes* were pulsed-field gel electrophoresis (PFGE) and multilocus sequence typing (MLST). These techniques lack the discriminatory power required for epidemiological surveillance (Moura et al. 2016, 2017). Genome-wide strain genotyping techniques (cgMLST) in *L. monocytogenes* were developed in 2016, providing the high discriminatory power required for epidemiological surveillance (Moura et al. 2016).

### Ruminant species affected

Regarding ruminant species, 74.7% of the cases (2120/2837) were reported in sheep, 14% (398/2837) in cattle, 1% (27/2837) in goats, 8.9% (253/2837) in sheep and goats, 1.3% (36/2837) in sheep and cattle and 0.1% (3/2837) in buffaloes (Table 2). These data and previous results from our group regarding the prevalence of *L. monocytogenes* in ruminants (Palacios-Gorba et al. 2021), would suggest that goats are more resistant to colonization and infection. The resistance of goats to listeriosis was also suggested by Viswanathan and Venkatarama Ayyar 1950 and Kumar et al. 2007 who reported that during outbreaks, goats were not affected, although they are reared together with infected sheep. The higher number of listeriosis outbreaks in sheep could reflect that they are more susceptible to infection. The high susceptibility of sheep to *Listeria* infection could also be observed in an outbreak caused by feeding contaminated silage (10^5^ Lm CFU/g), where ewes but not cows developed the disease (Wagner et al. 2005).

In buffalo, previous studies have reported the isolation *of L. monocytogenes*, evidence of infection, and measurable serological responses, indicating that this species may be susceptible to listeriosis and contribute to its epidemiology (Chaudhari et al. 2001; Barbuddhe et al. 2002). Other studies also revealed the presence of *Listeria* in the genital tract of buffalo (Shakuntala et al. 2006). Unfortunately, these studies in buffaloes did not meet the inclusion criteria and were not included in the statistical analysis.

### Silage implication

Ingestion of bad-quality silage has been traditionally considered the main route of infection for ruminants (Gray 1960; Gray and Schalm 1960; Low and Donachie 1997; Vazquez-Boland et al. 2001). A significant association between silage feeding and the development of *Listeria* outbreaks has been found (Wilesmith and Gitter 1986), and others have identified the silage as a putative contamination source of listeric outbreaks (Gray 1960; Grønstøl 1979; Low and Renton 1985; Nash et al. 1995; Ayars et al. 1999; Wagner et al. 2005) (Table 2). We state putative as, in these studies, no molecular characterization of the isolates was carried out to confirm that they were the same clones as those isolated from the organs of affected ruminants. Molecular characterization of the *L. monocytogenes* strains isolated from the silage and the organs of affected ruminants has previously been performed in three studies. With the molecular techniques available at the time of the study, Vázquez-Boland et al. 1992 first provided evidence of the epidemiologic link between silage consumption (1.9 × 10^6^*L. monocytogenes* CFU/g) and neurolisteriosis in ruminants. They found an identical phagotype and serotype in 2 silage samples and the brains of 3 affected sheep. Next, (Wiedmann et al. 1994) studied two outbreaks of *Listeria* encephalitis, one in sheep and one in goats, through random amplified polymorphic DNA (RAPD) patterns. Interestingly, the authors could find one strain with an identical RAPD pattern to the sheep brain isolate in the silage of the outbreak (4.8 × 10^5^*L. monocytogenes* CFU/g). However, in the goat outbreak (4.6 × 10^4^*L. monocytogenes* CFU/g) three brain isolates and one silage isolate were obtained, all with different random amplified polymorphic DNA patterns. Finally, Wiedmann et al. 1996 studied two additional outbreaks in dairy cattle using ribotyping as a molecular method. Only in one of the outbreaks matching ribotypes were found between silage and clinical isolates. Importantly, these studies used molecular techniques to investigate silage samples; however, other environmental samples were not analyzed as potential sources for *L. monocytogenes* infection. Moreover, it is questionable if the dose of *L. monocytogenes* to which ruminants were exposed in these 3 cases (1.9 × 10^6^ CFU/g, 4.8 × 10^5^ CFU/g, and 4.6 × 10^4^ CFU/g) would have been sufficient to cause clinical listeriosis. This is based on the fact that an experimental infection of ten sheep *per os* with a dose of 10^10^ CFU of *L. monocytogenes* (LCCN 95-962, serotype 1/2a obtained from an ewe with clinical listeriosis) was not sufficient to cause clinical listeriosis (Zundel and Bernard 2006).

It should be noted that a significant number of studies do not report silage feeding (Paterson 1940; Pounden et al. 1947; Thorp et al. 1947; Ferguson et al. 1951; Diplock and Mudgee 1957; Gitter et al. 1965; McDonald 1967; Wood 1972; Macleod et al. 1974; Du Toit 1977; Meredith and Schneider 1984; Reuter et al. 1989; Seaman et al. 1990; Johnson et al. 1996; Vandegraaff et al. 1981; Akpavie and Ikheloa 1992; Wiedmann et al. 1999; Şahin and Beytut 2006; Kumar et al. 2007; Prado et al. 2019; Osman et al. 2021) (note that in these studies it has to be considered that absence of reporting silage feeding does not necessarily imply that silage was not provided) or silage contamination with *L. monocytogenes* or *L. ivanovii* as a consistent finding in outbreaks of the disease (Løken 1982; Wardrope and MacLeod 1983; Green and Morgan 1994; Wiedmann et al. 1994; Bundrant et al. 2011; Dreyer et al. 2015; Fagundes et al. 2017). Interestingly, there are reports describing cases of listeriosis related to pasture grazing and not to contaminated silage (Fairley et al. 2012; Matto et al. 2017). Moreover, some studies (Fensterbank et al. 1984) used phage typing to show that the strains isolated from affected ruminants differed from those isolated from the silage. Similarly, pyrolysis mass spectrometry of *L. monocytogenes* isolates from a sheep listeriosis outbreak showed that the three available isolates from silage bales were differentiated from the three isolates obtained from the brains of affected sheep (Low et al. 1992). Accordingly, it was not possible to find matching *L. monocytogenes* isolates in silage and brain samples from two different outbreaks, one involving goats and using RAPD (Wiedmann et al. 1994) and one involving cattle and using ribotyping (Wiedmann et al. 1996). In 2016, García et al. 2016 reported an outbreak of enteric listeriosis in grazing steers supplemented with spoiled silage. In this report, two different strains of *L.* monocytogenes were isolated from clinical specimens and silage. *L. monocytogenes* serotype 1/2c was isolated from the gallbladder, and serotype 1/2b from the spoiled silage. More recently, whole genome sequencing revealed that three *L. monocytogenes* strains were responsible for an outbreak in cattle. It was confirmed that silage was the primary source of one strain, while the sources of the other two remained unidentified. (Whitman et al. 2020).

The evidence presented aligns with the assertion made by Walland et al. 2015 suggesting that the link between silage and listeriosis in ruminants should be considered due to the low number of systematic investigations on the one hand and divergent study results on the other. Previous studies have shown that *L. monocytogenes* was more prevalent in samples collected from feed bunks, water troughs, and bedding compared with the prevalence in feed or silage silos (Green and Morgan 1994; Mohammed et al. 2009; Palacios-Gorba et al. 2021). These results suggest that ruminants are more likely to be exposed to *L. monocytogenes* in their immediate environments than through silage (Mohammed et al. 2009). Additionally, there has been a suggestion that listeriosis in animals might even be transmitted through a venereal route (Wiedmann et al. 1999). Together, all these data would support previous reports that indicated that silage could be related to listeriosis outbreaks not by serving as a source of *Listeria* spp., but rather as a predisposing factor through an unknown mechanism of action (Gitter et al. 1986). Further characterization of these infection sources will contribute to controlling this disease.

Moreover, there is likely inter-host transmission over a relatively short period of time in the environment, particularly for those *L. monocytogenes* strains that are hypervirulent. It has been shown that as hypervirulent *L. monocytogenes* strains grow away from the host, they tend to reduce their SigB responsiveness and become less able to colonize new hosts (Hafner L et al. 2024). Furthermore, once CC1 is established somewhere, it seems to become entrenched, arguing that it stays in a farm environment (Moura et al. 2021). This would not be in favor of constantly importing new strains into ruminant farms, as silage would imply.

Due to the development of highly discriminatory typing techniques such as cgMLST, it is important that further studies are performed to determine the source of contamination during outbreaks in ruminants and finally analyze the distribution and variety of *L. monocytogenes* strains in silage and their relationship to isolates from clinical disease.

### Seasonality

Seasonal variations were observed in the reported outbreaks of listeriosis. In 52 outbreaks, information was provided regarding the season in which the listeriosis outbreak occurred (Table 2). The disease was more frequent in winter 46.2% (24/52), winter-spring 13.5% (7/52), late autumn/winter/spring 3.8% (2/52), and spring 19.2% (10/52). Altogether, 82.7% (43/52) of outbreaks were reported to occur during winter and spring.

The increased risk during the winter season has been traditionally associated with the seasonal feeding of silage (Low and Donachie 1997; Brugère-Picoux 2008; Dhama et al. 2015). However, as discussed in the previous section, there is no definitive proof of a molecular or epidemiological link between listeriosis and silage feeding in most cases. The higher number of listeriosis cases during colder months could also be explained by additional factors other than silage feeding, e.g. (1) *Listeria* can proliferate in environments where cold inhibits the growth of other bacteria (Brugère-Picoux 2008); (2) different husbandry types (Walland et al. 2015); (3) increased animal density during winter (Walland et al. 2015); (4) severe weather conditions, e.g. environmental contamination and nutritional stress after a period of continuous heavy rain and flooding of grazing pasture (Vandegraaff et al. 1981); (5) immunosuppression (Grønstøl and Øverås 1980); (6) tooth eruption (Barlow and McGorum 1985, (7) February and March coincide with late pregnancies among sheep population. Interestingly, the higher prevalence of described outbreaks in winter correlates with the prevalence of *L. monocytogenes* in ruminant feces, which peaked in winter (Husu 1990; Nightingale et al. 2005; Palacios-Gorba et al. 2021).

### Clinical syndromes, outbreak duration, and age

Apart from the localized forms which are beyond the scope of the current study, *L. monocytogenes* can cause five distinct syndromes in ruminants: encephalitis, abortion, diarrhea, septicemia and myelitis. Table 3 summarizes the clinical and pathological data provided by the 63 studies. The most common syndrome (clinical manifestation) reported was encephalitis (64.8%,1839/2837), followed by abortion (21.3%, 604/2837), diarrhea (9.2%, 260/2837), septicemia (6.7%, 191/2837) and myelitis (1.4%, 41/2837). These results are consistent with the results of two surveillance studies of ovine listeriosis in Scotland and England, where encephalitis cases were 2 to 5 times more common than abortion and enteric cases (Wilesmith and Gitter 1986; No authors listed 2018). The five syndromes do not tend to co-occur during the same outbreak. 70% (14/20) of reports where listerial abortion occurred showed that symptoms referable to the central nervous system were not observed. Similarly, 85.4% (35/41) of reports where neurolisteriosis cases occurred showed that abortions had not been detected. In 19.5% (554/2837) of the cases recorded the abortive and nervous types of listeriosis concurrently in the same farm and were described in both sheep and cattle (Smith 1955; Low and Renton 1985; Akpavie and Ikheloa 1992; Wagner et al. 2005; Osman et al. 2021). Importantly, ewes with neurolisteriosis had unaffected lambs. Moreover, a total absence of the abortive form was notable in encephalitic cases that occurred around the time of lambing (Reuter et al. 1989; Vázquez-Boland et al. 1992) whilst mothers suffering intrauterine infection had no encephalitis (McDonald 1967). These data would indicate that encephalitis of adult sheep and intrauterine infection and septicemia of lambs occur as separate entities (Dreyer et al. 2015). In addition, two reports described outbreaks where septicemia, abortions, and encephalitis were found in the same flock (Low and Renton 1985; Wagner et al. 2005). Septicemic cases (6.7%, 191/2837) were consistently observed in outbreaks together with diarrhea, encephalitic, or abortion cases. Diarrhea outbreaks were observed either with enteric symptoms (6.1%, 172/2837) or in combination with septicemic, nervous, and abortive manifestations (3.1%, 88/2837). Accordingly, epidemiological studies reported an overlap of the different clinical manifestations in only 9.3% (7/75) of affected flocks (Wilesmith and Gitter 1986). It is unclear why forms of listeriosis rarely overlap, and it is not clear whether genetic differences between *L. monocytogenes* strains may explain differences in this mutually exclusive presentation.

In the publications that met the inclusion criteria, *L. ivanovii* infection in ruminants was only associated with outbreaks of abortion in sheep but not cattle (Paterson 1940; Macleod et al. 1974; Dennis 1975; Sergeant et al. 1991; Chand and Sadana 1999; Şahin and Beytut 2006). However, outbreaks of abortion in cattle have also been described, but did not meet the inclusion criteria for the systematic review and meta-analysis (Alexander et al. 1992; Gill et al. 1997). *L. ivanovii* as a cause of abortion in sheep has been reported less frequently than *L. monocytogenes* (6 versus 14 reports) (Table 5).

Neurolisteriosis affects ruminants of all ages. Cases have been described in sheep and goats from 5 to 8 weeks old to 12 years old, in cattle from six months to 10 years old, and in buffaloes aged less than 40 days. Listeric abortion usually occurs in the last trimester and affects ruminants of all ages. Cases have been described, including ewes from more than 2 to 5 years old and cattle from 3 to 10 years old. Septicemia has mainly been reported in neonates. Diarrhea occurred in all ages (Table 3).

Since the contamination source could not be determined in most of the listeriosis outbreaks, the incubation time could not be calculated. The outbreak duration was 2 weeks to 8 months for encephalitis outbreaks, 1 to 2 months for abortion outbreaks, and 3 days for diarrhea cases.

The sheltering of aged, unproductive, chronically debilitated and abandoned cattle in traditional cow shelters (gaushalas) is an ancient practice in India. Recent studies highlight that animals housed in gaushalas may play a relevant role in the epidemiology of listeriosis. In particular, high listeriolysin O seropositivity was observed among cattle from the gaushala when compared with an organized farm (Ramanjeneya et al. 2019). Management practices within cattle shelters—often characterized by overcrowding, limited biosecurity measures, and heterogeneous health status—may further favor the maintenance and dissemination of *Listeria* species (Sharma et al. 2020). These findings support the need to consider livestock shelters and similar animal populations in epidemiological frameworks and encourage further research to clarify their role as silent carriers or reservoirs.

### Description of morbidity and case fatality data

Upon analysis of the data collected from the articles selected for this study (Table 4), the mean and 95% confidence interval (in parenthesis) of the morbidity (proportion of ruminants affected by listeriosis within the herd population over the studied period) in ruminants suffering from the abortive form of infection was 7.3% (2–12.5), whilst for the CNS and diarrheal forms this was 7.7% (0–15.8) and 7.9% (4.7–11.1) respectively.

With regards to the case fatality rate, we observed that the mean and 95% confidence interval in animals suffering from the abortive form of the disease was 0.5% (0–1.9). The CNS and diarrheal forms of the disease demonstrated relatively higher case fatality rates of 83.1% (71.2–94.9) and 41.7% (29.2–54.1), respectively.

The limited number of data points for the septicemic form of the disease results in a high margin of error and inaccuracy for the calculation of confidence intervals for both morbidity and case fatality proportions and was therefore not calculated.

**Figure 2.**
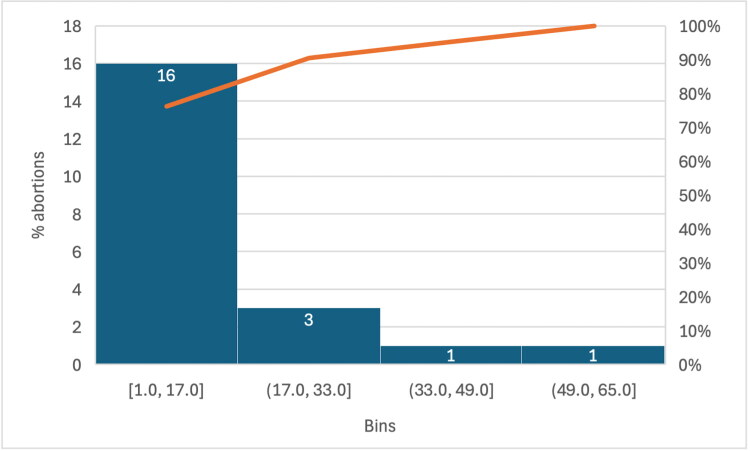
Abortion rates Proportion of abortions amongst females suffering from the abortive form of the disease grouping the data into bins of 16%, the Pareto line is shown in orange.

Regarding the proportion of abortions amongst females suffering from the abortive form of the disease, the analysis revealed a mean of 12.8 (95% CI of 7.1–18.5). The included studies reported abortion rates ranging from 1 to 50%. Grouping the data into bins of 17% (Figure 2), we demonstrate that the majority (90%) of studies report an abortion rate of 1–17%. A further 15% of studies reported an abortion rate of between 17 and 33%, whilst very few studies report higher abortion rates of 33–49% (5% of studies) and 49–65% respectively (5% of studies). The Pareto line (orange, Figure 2) demonstrates the accumulated proportion of studies across the defined bins.

### Meta-analysis

**Figure 3.**
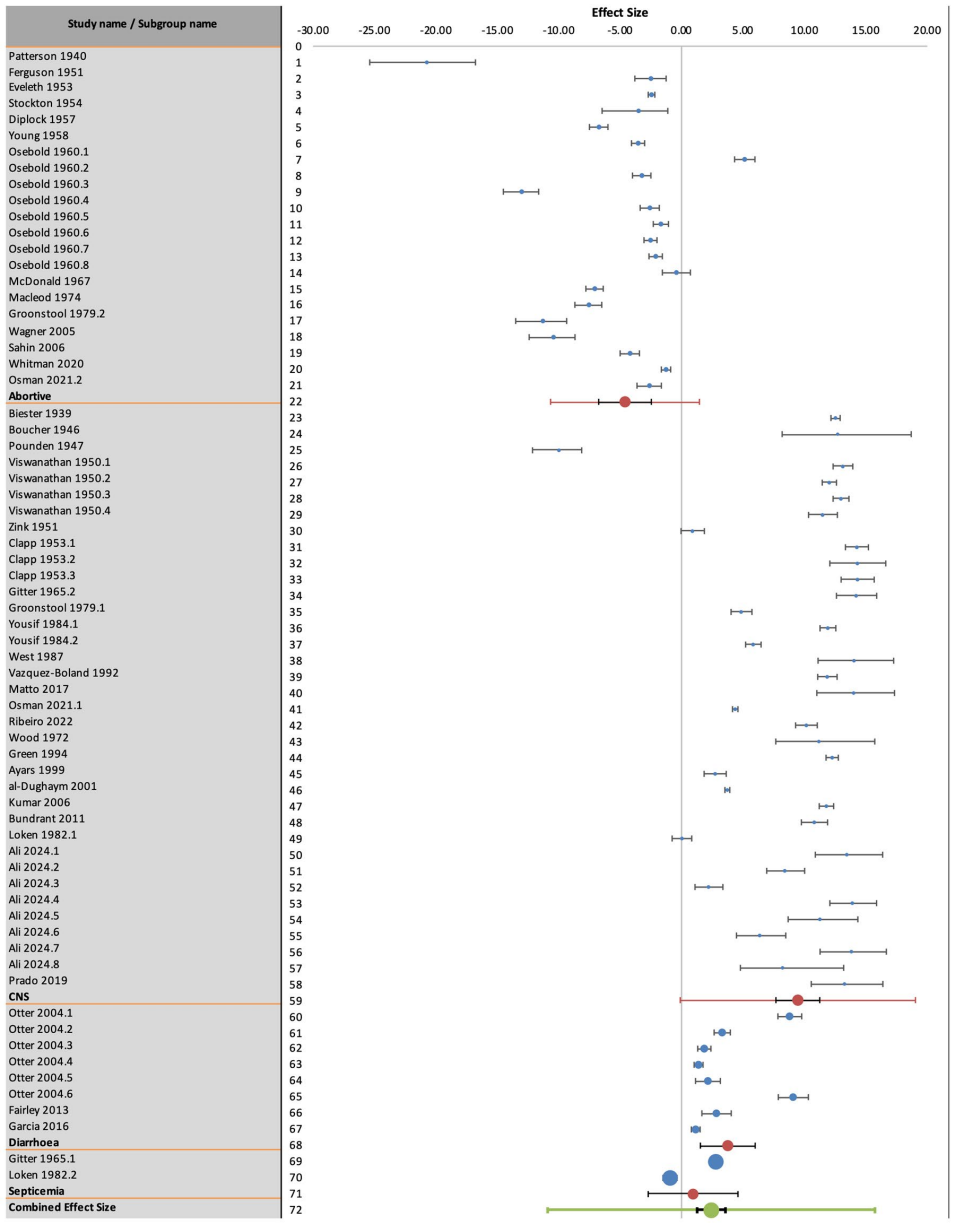
Forest Plot showing the overall and clinical manifestation-specific Hedges’ g statistic (the bias-adjusted standardized mean difference between morbidity and case fatality rate for each study). Hedges’ g values below 1 are summary indicators that morbidity > case fatality, whilst values above 1 indicate that case fatality > morbidity across the included studies. A value of 0 indicates that there was no difference between the proportion of morbidity and case fatality among the tested studies.

The meta-analysis demonstrates that the calculated summary statistic (Hedges’ g) varies considerably between the clinical characteristics analyzed (Supplementary Table 2). The mean Hedges’ g for the ‘abortive’ infection type was −4.60 (favoring morbidity), whilst the values for CNS infections, diarrheal and septicemic infections were 9.46, 3.76 and 0.94 respectively (favoring case-fatality; Supplementary Table 2) (Figure 3). Abortive infections, therefore, can be observed to be concurrent with high morbidity and low case maternal fatality. The highest proportional case fatality was observed in CNS infections, followed by diarrheal infection, and finally septicemic infection (although the number of studies in this group was very low) (Figure 3).

The overall I^2^ statistic was 99.66%, suggesting a very high degree of heterogeneity across the included studies. When analyzed by subgroup, this improved slightly, although the heterogeneity remained very high. The I^2^ statistic for studies describing abortive infections was 98.3%, whilst that for studies reporting CNS, diarrheal, and septicemic infections was 99.4%, 98.1%, and 99.2%, respectively. The high degree of calculated heterogeneity amongst the included studies resulted in a broad prediction interval, both in the global analysis as in subgroup analysis. As a result of the high heterogeneity, the calculated mean effect sizes did not achieve statistical significance.

**Figure 4.**
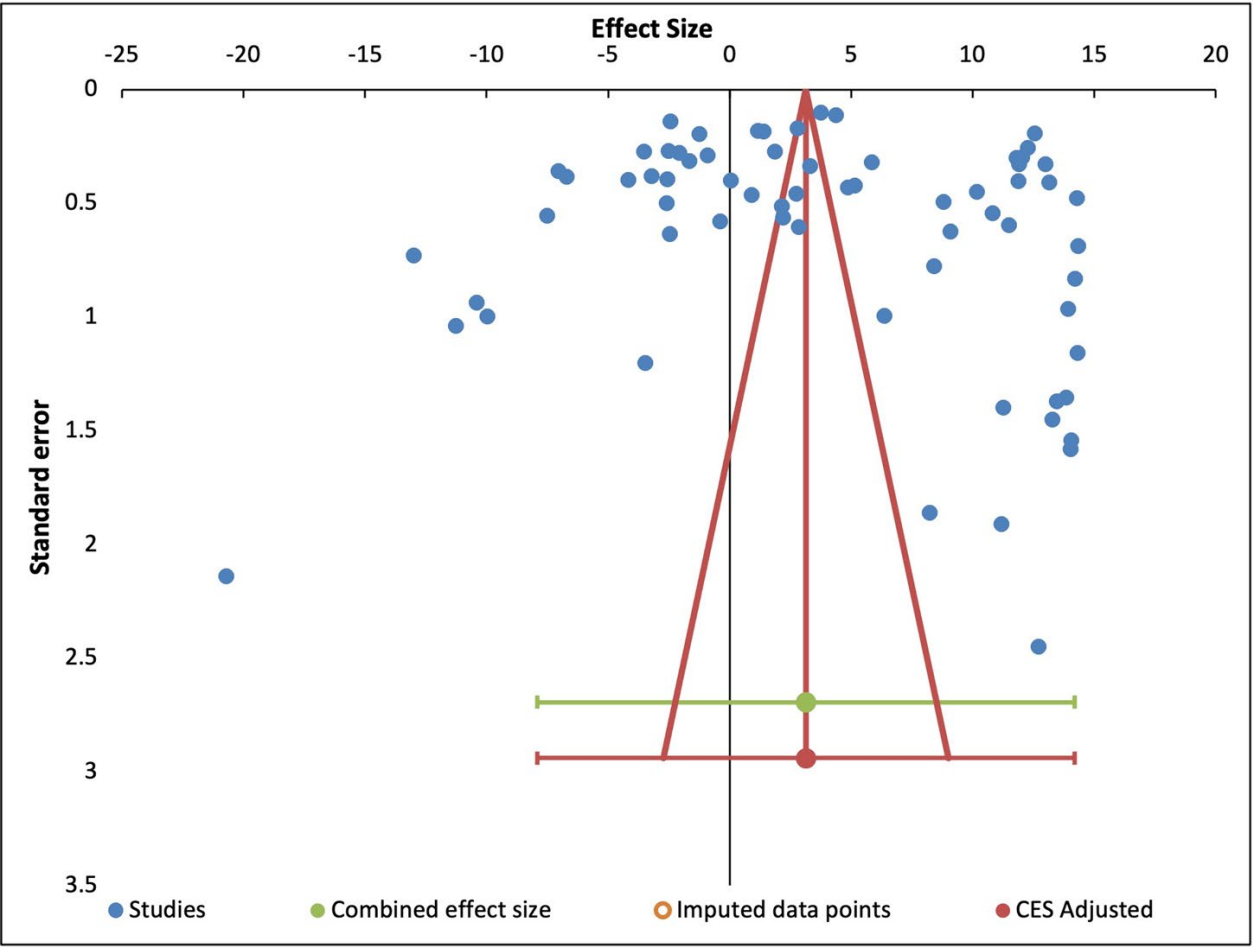
Funnel plot showing the dispersion and heterogeneity of included studies.

The analysis of possible publication bias (Figure 4) shows a broad distribution to the left and right side of the mean combined effect size. The high degree of heterogeneity of the included studies makes it difficult to draw a meaningful conclusion from the data. It can be seen clearly that there are four outlier studies (upper left-hand quadrant), and one study that appears to be an extreme outlier, situated in the lower left quadrant of the plot. The majority of studies show a low standard error and a broad distribution of effect sizes above and below the mean adjusted combined effects value (vertical red line). Few included studies fit within the predicted limits of the adjusted combined effect size, supporting the previous observation that there exists a high degree of variability and heterogeneity within the study dataset. Our analysis was not able to predict missing studies, which would have provided a more balanced coverage of the research. We predict a moderate to high level of publication bias for this study.

### Fever, clinical pathology, and lesions

Body temperature was recorded in 28/63 of the studies included in this review (Table 3). Fever was reported in 31.3%–46.8% of patients (Braun et al. 2002; Schweizer et al. 2006). 21.4% (6/28) of the studies, including cattle and small ruminants, reported no fever. Results of blood testing indicate that leukocytosis is not a consistent disease feature (Table 3). Only eight studies reported white blood cell counts during listeriosis cases in ruminants, mainly in reports describing neurolisteriosis cases (88.9%, 8/9). There is only one report regarding a *Listeria* abortion outbreak that provides this clinical parameter (Young and Firehammer 1958). In this case, leukocytosis was only present in 6.2% (1/16) of the cattle. Regarding leukocytosis in neurolisteriosis cases in cattle, two studies of individual animals showed that there was no leukocytosis (Cole 1946; Oshima 1974), and another study involving 94 cattle reported leukocytosis in 43.6% (41/94) of animals (Schweizer et al. 2006). In the case of neurolisteriosis cases in small ruminants, leukocytosis was described in 66.7% (2/3), 22.2% (2/9), and 8% (5/62) of the animals (Wood 1972; Wardrope and MacLeod 1983; Braun et al. 2002).

Analysis of cerebrospinal fluid (CSF) in neurolisteriosis cases, showed significantly elevated white blood cell counts in 77% (57/74) of affected cows (Schweizer et al. 2006) and in 81% (17/21) − 88.9% (8/9) of affected sheep and goats (Scott 1993; Braun et al. 2002). Increased CSF protein concentration was found in 69% (51/74) (Schweizer et al. 2006) of cattle and in 86% (18/21) − 100% (9/9) cases of sheep and goats (Scott 1993; Braun et al. 2002) affected by neurolisteriosis. Compared to humans, *L. monocytogenes* isolation from CSF fails in up to 90% of ruminant cases (Bagatella et al. 2022).

These data suggest that total blood leucocyte count has little diagnostic value since leukocytosis has not been found to be a feature of listeriosis in ruminants. However, leucocyte count in the CSF may be helpful for diagnosis in neurolisteriosis cases.

### Lesions

Whilst histopathological lesions are commonly observed in all forms of the disease, not all the studies described gross pathological lesions in animals suffering from listeriosis (Table 3). In encephalitis cases, brain macroscopic lesions are frequent (43.3%, 13/30), and congestion, hemorrhages, malacia, and clouding of CSF are the most common lesions. In ruminants, the brainstem is specifically targeted, and the most common histopathological findings are micro-abscesses in the brainstem, perivascular lymphocytic cuffing, meningitis, and gliosis. In the abortive form, there is placentitis and endometritis, as well as multiple necrotic foci in the liver and spleen in the fetuses that are often autolytic, and clear to sanguineous fluid in the body cavities, with 61.5% (8/13) of the reports describing macroscopic lesions. Clinical enteric listeriosis is associated with abomasitis and enteritis, with 100% (7/7) of the reports describing gross lesions in these organs. In the septicemic form, pyogranulomas may be observed in different organs, with 100% (4/4) of the reports describing gross lesions.

### Treatment efficacy

Acquired antimicrobial resistance in *L. monocytogenes* is rare (2.23% isolates) and is mainly towards tetracyclines (mostly due to *tetM*), trimethoprim (*dfrD*), lincosamides (*lnuG*), macrolides (*ermB*, *mphB*), and phenicols (*fexA*) (Markovich et al. 2024; Moura et al. 2024). In humans, the standard therapy for listeriosis is based on amoxicillin or ampicillin in combination with gentamicin (Baquero et al. 2020). Excluding the reports describing abortive cases (of which none quantify the success rate of the antibiotic treatment), only 11 studies related to septicemia or encephalitic cases that include more than five ruminants reported the results of antibiotic treatment (Table 5). Regarding septicemia, one study used penicillin and oxytetracycline for lambs with no success (none of the 60 lambs recovered, (Gitter et al. 1965) and another study in goats used penicillin with 100% survival (10/10 goats survived, Løken and Grønstøl 1982).

Regarding encephalitic cases in cattle, Bundrant et al. 2011 reported poor response to treatment (50% (2/4) survived when treated with florfenicol, and 0% (0/5) survived after receiving a course of ampicillin), whilst a better response was reported by Ayars et al. 1999 (80% (4/5) bulls recovered after oxytetracycline treatment) and Schweizer et al. 2006 (recovery rates of 78.1% (25/32) for gentamicin plus amoxicillin plus flunixin, 76.2% (16/21) for oxytetracycline, 55.5% (10/18) for chloramphenicol, 60% (6/10) for penicillin G, 80% (4/5) for amoxicillin and 100% (1/1) for florfenicol).

There are significant differences in results of studies cases in sheep. Meredith and Schneider (1984) and Vázquez-Boland et al. (1992) reported survival rates of 0% (0/10) and 5.7% (3/53) using chloramphenicol or a combination of tetracycline, penicillin, gentamicin, and spiramycin. In contrast, Scott (1993) reported a 24% (5/21) survival by using penicillin Green and Morgan (1994) described a 37.5% (3/8) survival by using oxytetracycline, and Braun et al. 2002 reported survival of 100% (1/1) with penicillin, 66.7% (6/9) with gentamicin-ampicillin, 18.2% (2/11) with oxytetracycline, and 6.7% (1/15) with chloramphenicol. Altogether, these results show that the mortality rate remains high in ruminants, as in humans, despite the availability of treatment options.

In cattle, anti-inflammatory agents did not influence the survival rate (68.9% (31/45) survivors in the group that received anti-inflammatory medication versus 63.2% (31/49) survivors in the group that did not receive anti-inflammatory medication) (Schweizer et al. 2006). Corticosteroids probably harm only the most immunosuppressed in humans. Hence possibly explaining why it may have no effect in cattle, which are not expected to be immunosuppressed. Considering that the administration of dexamethasone increased the shedding of *L. monocytogenes* in the milk of cows (Wesley et al. 1989), the use of corticosteroid treatments during listeriosis outbreaks is not advisable.

### Genetic characteristics

*L. monocytogenes* strain characterization was performed only in 14 of the 63 publications that met the inclusion criteria and identified *L. monocytogenes* as the causative agent. Eleven of these publications used serotyping characterization, one used ribotyping (Bundrant et al. 2011), one used multilocus sequence typing (MLST) and pulsed-field gel electrophoresis (PFGE) (Dreyer et al. 2015), whilst another study used whole-genome sequencing (Whitman et al. 2020). Since serotyping allows the differentiation of only 13 serotypes, it has limited value for epidemiological tracking, whereas WGS and cgMLST are the most powerful tools to link clinical, food, and environmental isolates in epidemiological surveillance. Five out of 11 ruminant outbreaks reported the presence of serotype 4b, which is the preponderant serotype in major human listeriosis sporadic cases and outbreaks and ruminant neurolisteriosis cases, as it contains the hypervirulent clonal complexes CC1, CC4, and CC6 (Maury et al. 2016, 2019; Bagatella et al. 2022). These data indicate that there is a lack of studies reporting listeriosis sporadic cases and outbreaks in ruminants that performed genetic characterization of the isolates since the advent of more precise molecular techniques such as the MLST (Salcedo et al. 2003; Ragon et al. 2008) or the cgMLST (Moura et al. 2016) methods.

Cardenas-Alvarez et al. (2022); Dreyer et al. (2016) and Papić et al. (2019) analyzed different collections of ruminant listeriosis isolates from the United Kingdom, Switzerland, Europe, and the USA recovered from 1996 to 2020, by MLST or whole-genome sequencing. Ruminant rhombencephalitis and fetal infections isolates were significantly overrepresented in lineage I which is also strongly associated with a clinical origin in humans (Maury et al. 2016). It would be interesting to confirm these results by screening a larger number of strains from ruminant listeriosis outbreaks in other countries. Due to the scarcity of genetic data from ruminant cases, more research is needed to determine whether identical strains cause CNS infections in ruminants and humans. The incorporation of genotypic information of *L. monocytogenes* isolates from animal outbreaks will facilitate global epidemiological surveillance.

Notably, a recent ovine listeriosis outbreak in China was caused by a hybrid sub-lineage of *L. monocytogenes* comprising hypervirulent isolates that acquired a partial LIPI-2 (*Listeria* Pathogenicity Island 2) from *L. ivanovii* (Yin et al. 2019). The intriguing finding of a newly described hypervirulent hybrid *L. monocytogenes* in an ovine listeriosis outbreak in China indicates that ruminant surveillance is critical.

### Limitations

There are some limitations relating to this review. The quality and reported characteristics of the included research varied greatly, which limited the total number of studies in each meta-analysis. There was little consistency in isolation and typing methods for strain identification, as well as a lack of studies representing clinical subtypes.

## Conclusions

Our systematic review and meta-analysis examined the epidemiology, clinical and pathological characteristics, outcomes, and efficacy of antibiotic treatment of ruminants suffering from listeriosis. This review highlights the need to increase and report on epidemiological research into ruminant listeriosis worldwide. Such research should incorporate whole genome sequencing in order to improve our understanding of listeriosis, from its origin on farms to its clinical outcomes in hospitals, thereby enhancing animal and human health and food safety. Here we analyzed the available data on clinical and pathological parameters during listeriosis in ruminants and the conclusions obtained could help clinicians diagnose listeriosis in ruminants, decide whether to proceed with treatment or supportive therapy, and know the prognosis of listeriosis. Moreover, we highlight the importance of animal surveillance in reducing the environmental spread of *L. monocytogenes* and, ultimately, human clinical cases. The main conclusions of this work are:

1. Due to the lack of active surveillance data and the fact that outbreak investigations are not routinely performed in ruminant farms, the true incidence of listeriosis in ruminants worldwide is unknown. It is possible that *Listeria* infections in domestic animals are frequent worldwide and that the lack of reports in many countries indicates a lack of investigation rather than the absence of disease. Geographic gaps exist in the data on listeriosis cases in ruminants, especially in South America, Europe, Africa and Asia. It is necessary to increase the number of studies and reports of listeriosis in animals to improve our understanding of animal listeriosis, better treat this deadly animal disease, and improve public health.
2. Despite large-scale and systematic analysis integrating comparative genomics and epidemiological data that has been performed in human listeriosis outbreaks, this analysis of ruminant listeriosis outbreaks is lacking. Consequently, the general understanding of listeriosis outbreaks in domestic ruminants is based on a limited number of studies on small outbreaks in cattle and small ruminants. Consequently, there is a need to examine the genomic and virulence characteristics of the ruminant isolates that cause outbreaks.
3. 88.9% (56/63) of the studies related to ruminant outbreaks were conducted before 2016, and consequently, genome-wide typification techniques with high discriminatory power for epidemiological surveillance could not applied (e.g. WGS and cgMLST).
4. The incidence of listeriosis cases in ruminants appears to be higher during the colder seasons than at any other time of year.
5. Other risk factors than silage feeding should be considered, and they may play an important role in the epidemiology of listeriosis in ruminants. Further research should focus on the infection source and the infectious dose necessary for ruminants to develop clinical symptoms.
6. Regarding species, sheep seem more susceptible to infection than cows, while goats seem more resistant to colonization and infection.
7. *L. monocytogenes* causes five main distinct syndromes in ruminants: diarrhea, septicemia, encephalitis, abortion, and myelitis, which do not tend to co-occur during the same outbreak.
8. Overall, mean morbidity is identical in the abortive and encephalitic forms of the disease, whilst, in contrast, the encephalitic, diarrheal and septicemic forms are associated with elevated mean case fatality rates.
9. The majority of studies describing abortive disease outbreaks reported an abortion rate between 1 and 17%.
10. This meta-analysis is the first one in this field to statistically analyze the morbidity and case fatality of the different forms of the disease. The key conclusions are that the calculated Hedge’s g statistic confirms differences in the morbidity and case fatality rates between the disease forms, although the results were not found to be statistically significant as a result of the high heterogeneity of included studies.
11. We interpret the possibility of a moderate-to-high publication bias in the literature, as our analysis revealed a possible absence of studies with smaller effect sizes and variances. This is common and expected in meta-analyses of observational studies.
12. Ruminant listeriosis is characterized by a poor response to antibiotic treatment as in humans. Much uncertainty remains in understanding the sequelae of *L. monocytogenes* infection and recovery from these conditions over time. More studies also are needed to assess the treatment effects of antibiotics and corticosteroids on infection recovery.
13. Fever and blood leukocytosis are not a constant finding during listeriosis. However, leucocyte count in the CSF could be diagnostically helpful in neurolisteriosis cases.
14. Whilst histopathological lesions are commonly observed in all forms of the disease, gross pathological lesions in ruminants suffering from listeriosis are not a constant finding.
15. *L. ivanovii* infection in ruminants has only been associated with abortion outbreaks in sheep, but not in cattle.

## Supplementary Material

Supplementary_Table_1_and_2_revclean.docx
