# Supplementary material for: Epidemiology, clinical and pathological features and outcomes of listeriosis in ruminants: a systematic review and meta-analysis
Source: Vet Q. 2025 Dec 8;45(1):2598257. doi: 10.1080/01652176.2025.2598257 (PMC12690773; doi:10.1080/01652176.2025.2598257)
Supplement: Supplementary_Table_1_and_2_revclean.docx [file TVEQ_A_2598257_SM6910.docx]

**Supplementary Table 1a: JBI Critical Appraisal Checklist for Case Report**

| Study / Question | Q1 | Q2 | Q3 | Q4 | Q5 | Q6 | Q7 | Q8 |
| --- | --- | --- | --- | --- | --- | --- | --- | --- |
| Graham 1938 | N | UN | Y | Y | U | Y | NA | Y |
| Biester 1939 | UN | UN | Y | UN | NA | NA | NA | Y |
| Paterson 1940 | Y | Y | Y | Y | NA | NA | NA | Y |
| Harbour 1941 | N | Y | Y | Y | N | NA | NA | Y |
| Cole 1946 | N | UN | Y | UN | UN | NA | NA | Y |
| Boucher 1946 | UN | Y | Y | N | Y | NA | NA | Y |
| Thorp 1947 | UN | Y | UN | UN | NA | NA | NA | Y |
| Pounden 1947 | Y | Y | Y | UN | Y | Y | NA | Y |
| Ferguson 1951 | UN | UN | UN | Y | UN | NA | NA | Y |
| Zink 1951 | UN | Y | Y | Y | Y | Y | NA | Y |
| Eveleth 1953 | N | N | N | N | UN | UN | NA | Y |
| Clapp 1953 | UN | N | UN | N | UN | UN | NA | Y |
| Stockton 1954 | UN | UN | UN | Y | NA | NA | NA | Y |
| Smith 1955 | UN | Y | Y | Y | Y | Y | NA | Y |
| Diplock 1957 | UN | UN | UN | UN | UN | UN | NA | Y |
| Young 1958 | Y | Y | Y | Y | Y | Y | NA | Y |
| Osebold 1960 | UN | Y | UN | Y | UN | UN | NA | Y |
| Gitter 1965 | UN | Y | UN | Y | Y | Y | NA | Y |
| Gates 1967 | UN | UN | Y | UN | NA | NA | NA | Y |
| McDonald 1967 | Y | Y | UN | Y | NA | NA | NA | Y |
| Wood 1972 | UN | Y | Y | UN | Y | Y | NA | Y |
| Macleod 1974 | Y | Y | UN | Y | Y | UN | NA | Y |
| Oshima 1974 | UN | Y | UN | UN | UN | UN | NA | UN |
| du Toit 1977 | Y | UN | UN | Y | N | NA | NA | Y |
| Groonstool 1979 | Y | Y | UN | UN | UN | UN | NA | Y |
| Vandegraaff 1981 | UN | UN | UN | Y | NA | NA | NA | Y |
| Price 1981 | N | UN | Y | UN | Y | Y | NA | UN |
| Loken 1982 | Y | Y | Y | Y | Y | Y | NA | Y |
| Wardrope 1983 | Y | Y | Y | Y | UN | NA | NA | Y |
| Yousif 1984 | UN | Y | Y | Y | UN | UN | NA | Y |
| Meredith 1984 | UN | UN | UN | Y | UN | UN | NA | Y |
| Low 1985 | Y | Y | Y | Y | UN | Y | NA | Y |
| West 1987 | UN | UN | UN | N | Y | UN | NA | Y |
| Reuter 1989 | UN | UN | UN | UN | UN | UN | NA | Y |
| Seaman 1990 | Y | Y | Y | Y | UN | UN | NA | Y |
| Sergeant 1991 | UN | Y | Y | Y | UN | UN | NA | Y |
| Vazquez-Boland 1992 | Y | Y | Y | Y | Y | Y | NA | Y |
| Akpavie 1992 | UN | Y | Y | Y | UN | Y | NA | Y |
| Nash 1995 | Y | Y | UN | UN | UN | UN | NA | Y |
| Chand 1999 | UN | UN | Y | Y | UN | UN | NA | Y |
| Ayars 1999 | UN | UN | Y | Y | Y | UN | NA | Y |
| al-Dughaym 2001 | UN | UN | Y | Y | UN | UN | NA | Y |
| Wagner 2005 | Y | Y | Y | Y | Y | Y | NA | Y |
| Sahin 2006 | UN | UN | UN | Y | UN | UN | NA | Y |
| Kumar 2006 | UN | UN | Y | Y | UN | UN | NA | Y |
| Bundrant 2011 | N | Y | Y | Y | Y | Y | NA | Y |
| Fairley 2013 | UN | UN | UN | Y | UN | UN | NA | Y |
| Dreyer 2015 | Y | Y | Y | Y | NA | NA | NA | Y |
| Garcia 2016 | UN | Y | Y | Y | UN | UN | NA | Y |
| Matto 2017 | UN | UN | UN | Y | Y | Y | NA | Y |
| Prado 2019 | Y | Y | UN | Y | Y | Y | NA | Y |
| Whitman 2020 | Y | UN | Y | Y | Y | Y | NA | Y |
| Osman 2021 | UN | UN | Y | Y | Y | Y | NA | Y |
| Ribeiro 2022 | Y | UN | Y | Y | Y | UN | NA | Y |
| Ali 2024 | Y | UN | Y | Y | NA | NA | NA | Y |

Yes (Y), No (N), Unclear (UN) and Not Applicable (NA), Question 1- 10 (Q1-Q10)

Q1. Were patient’s demographic characteristics clearly described?

Q2. Was the patient’s history clearly described and presented as a timeline?

Q3. Was the current clinical condition of the patient on presentation clearly described?

Q4. Were diagnostic tests or assessment methods and the results clearly described?

Q5. Was the intervention(s) or treatment procedure(s) clearly described?

Q6. Was the post-intervention clinical condition clearly described?

Q7. Were adverse events (harms) or unanticipated events identified and described?

Q8. Does the case report provide takeaway lessons?

**Supplementary Table 1b: JBI Critical Appraisal Checklist for Cohort Studies**

| Study / Question | Q1 | Q2 | Q3 | Q4 | Q5 | Q6 | Q7 | Q8 | Q9 | Q10 | Q11 |
| --- | --- | --- | --- | --- | --- | --- | --- | --- | --- | --- | --- |
| Scott 1993 | Y | Y | Y | N | N | Y | N | Y | Y | Y | Y |
| Green 1994 | Y | Y | Y | Y | Y | Y | Y | Y | Y | Y | Y |

Yes (Y), No (N), Unclear (UN) and Not Applicable, Question 1- 11 (Q1-Q11)

Q1. Were the two groups similar and recruited from the same population?

Q2. Were the exposures measured similarly to assign people to both exposed and unexposed groups?

Q3. Was the exposure measured in a valid and reliable way?

Q4. Were confounding factors identified?

Q5. Were strategies to deal with confounding factors stated?

Q6. Were the groups/participants free of the outcome at the start of the study (or at the moment of exposure)?

Q7. Were the outcomes measured in a valid and reliable way?

Q8. Was the follow up time reported and sufficient to be long enough for outcomes to occur?

Q9. Was follow up complete, and if not, were the reasons to loss to follow up described and explored?

Q10. Were strategies to address incomplete follow up utilized?

Q11. Was appropriate statistical analysis used?

**Supplementary Table 1c: JBI Critical Appraisal Checklist for Cases Series**

| Study / Question | Q1 | Q2 | Q3 | Q4 | Q5 | Q6 | Q7 | Q8 | Q9 | Q10 |
| --- | --- | --- | --- | --- | --- | --- | --- | --- | --- | --- |
| Viswanathan 1950 | Y | Y | Y | UN | UN | Y | UN | UN | Y | NA |
| Dennis 1975 | UN | Y | Y | Y | UN | N | UN | UN | N | NA |
| Braun 2002 | Y | Y | Y | Y | Y | N | Y | N | N | NA |
| Clark 2004 | Y | Y | Y | Y | Y | N | N | N | N | NA |
| Otter 2004 | Y | Y | Y | Y | Y | Y | UN | UN | UN | NA |
| Schweizer 2006 | Y | Y | Y | Y | Y | N | Y | UN | N | NA |

Yes (Y), No (N), Unclear (UN) and Not Applicable, Question 1- 10 (Q1-Q10)

Q1. Were there clear criteria for inclusion in the case series?

Q2. Was the condition measured in a standard, reliable way for all participants included in the case series?

Q3. Were valid methods used for identification of the condition for all participants included in the case series?

Q4. Did the case series have consecutive inclusion of participants?

Q5. Did the case series have complete inclusion of participants?

Q6. Was there clear reporting of the demographics of the participants in the study?

Q7. Was there clear reporting of clinical information of the participants?

Q8. Were the outcomes or follow up results of cases clearly reported?

Q9. Was there clear reporting of the presenting site(s)/clinic(s) demographic information?

Q10. Was statistical analysis appropriate?

**Supplementary Table 2**: Calculated summary effect size meta-analysis data, analyzed by subgroup (clinical manifestation of the disease). Hedges’ g values of below 1 favor morbidity whilst above 1 favor case fatality. The weighting was calculated according to the population size. Additionally, I^2^, T^2^ and T values were calculated to estimate heterogeneity. The confidence interval (CI) upper and lower limits of the summary effect size as well as the upper and lower limits of the prediction interval (PI) are also calculated and presented.

| **Study name / Subgroup name** | **Hedges' g** | **CI Lower limit** | **CI Upper limit** | **Weight** | **I^2^** | **T^2^** | **T** | **PI Lower limit** | **PI Upper limit** |
| --- | --- | --- | --- | --- | --- | --- | --- | --- | --- |
| Patterson 1940 | -20,73 | -25,04 | -16,43 | 3,10% |  |  |  |  |  |
| Ferguson 1951 | -2,49 | -3,76 | -1,22 | 4,79% |  |  |  |  |  |
| Eveleth 1953 | -2,44 | -2,71 | -2,17 | 5,04% |  |  |  |  |  |
| Stockton 1954 | -3,50 | -6,17 | -0,83 | 4,22% |  |  |  |  |  |
| Diplock 1957 | -6,72 | -7,48 | -5,97 | 4,95% |  |  |  |  |  |
| Young 1958 | -3,53 | -4,07 | -2,99 | 5,00% |  |  |  |  |  |
| Osebold 1960.1 | 5,13 | 4,31 | 5,96 | 4,93% |  |  |  |  |  |
| Osebold 1960.2 | -3,23 | -3,98 | -2,48 | 4,95% |  |  |  |  |  |
| Osebold 1960.3 | -13,01 | -14,45 | -11,57 | 4,71% |  |  |  |  |  |
| Osebold 1960.4 | -2,58 | -3,36 | -1,80 | 4,94% |  |  |  |  |  |
| Osebold 1960.5 | -1,68 | -2,30 | -1,07 | 4,98% |  |  |  |  |  |
| Osebold 1960.6 | -2,52 | -3,05 | -2,00 | 5,00% |  |  |  |  |  |
| Osebold 1960.7 | -2,10 | -2,65 | -1,56 | 5,00% |  |  |  |  |  |
| Osebold 1960.8 | -0,04 | -1,18 | 1,10 | 4,83% |  |  |  |  |  |
| McDonald 1967 | -7,06 | -7,77 | -6,36 | 4,96% |  |  |  |  |  |
| Macleod 1974 | -7,54 | -8,63 | -6,44 | 4,85% |  |  |  |  |  |
| Groonstool 1979.2 | -11,29 | -13,36 | -9,21 | 4,40% |  |  |  |  |  |
| Wagner 2005 | -10,42 | -12,29 | -8,56 | 4,51% |  |  |  |  |  |
| Sahin 2006 | -4,19 | -4,98 | -3,41 | 4,94% |  |  |  |  |  |
| Whitman 2020 | -1,26 | -1,64 | -0,88 | 5,02% |  |  |  |  |  |
| Osman 2021.2 | -2,61 | -3,60 | -1,62 | 4,88% |  |  |  |  |  |
| **Abortive** | **-4,59** | **-6,74** | **-2,44** | **25,27%** | **98,35%** | **7,27** | **2,70** | **-10,66** | **1,49** |
| Biester 1939 | 12,53 | 12,16 | 12,90 | 2.89% |  |  |  |  |  |
| Boucher 1946 | 12,70 | 7,45 | 17,96 | 2.26% |  |  |  |  |  |
| Pounden 1947 | -9,98 | -11,98 | -7,98 | 2.77% |  |  |  |  |  |
| Viswanathan 1950.1 | 13,12 | 12,32 | 13,92 | 2.87% |  |  |  |  |  |
| Viswanathan 1950.2 | 12,02 | 11,44 | 12,60 | 2.88% |  |  |  |  |  |
| Viswanathan 1950.3 | 12,97 | 12,32 | 13,61 | 2.88% |  |  |  |  |  |
| Viswanathan 1950.4 | 11,47 | 10,30 | 12,64 | 2.85% |  |  |  |  |  |
| Zink 1951 | 0,88 | -0,07 | 1,83 | 2.87% |  |  |  |  |  |
| Clapp 1953.1 | 14,26 | 13,33 | 15,20 | 2.86% |  |  |  |  |  |
| Clapp 1953.2 | 14,31 | 12,03 | 16,59 | 2.72% |  |  |  |  |  |
| Clapp 1953.3 | 14,32 | 12,97 | 15,66 | 2.83% |  |  |  |  |  |
| Gitter 1965.2 | 14,21 | 12,57 | 15,84 | 2.80% |  |  |  |  |  |
| Groonstool 1979.1 | 4,85 | 4,00 | 5,70 | 2.87% |  |  |  |  |  |
| Yousif 1984.1 | 11,90 | 11,26 | 12,54 | 2.88% |  |  |  |  |  |
| Yousif 1984.2 | 5,82 | 5,19 | 6,45 | 2.88% |  |  |  |  |  |
| West 1987 | 14,03 | 10,96 | 17,11 | 2.61% |  |  |  |  |  |
| Vazquez-Boland 1992 | 11,85 | 11,07 | 12,64 | 2.87% |  |  |  |  |  |
| Matto 2017 | 14,00 | 10,84 | 17,16 | 2.59% |  |  |  |  |  |
| Osman 2021.1 | 4,37 | 4,15 | 4,59 | 2.89% |  |  |  |  |  |
| Ribeiro 2022 | 10,15 | 9,27 | 11,04 | 2.87% |  |  |  |  |  |
| Wood 1972 | 11,17 | 7,14 | 15,20 | 2.47% |  |  |  |  |  |
| Green 1994 | 12,26 | 11,76 | 12,76 | 2.89% |  |  |  |  |  |
| Ayars 1999 | 2,73 | 1,83 | 3,63 | 2.87% |  |  |  |  |  |
| al-Dughaym 2001 | 3,73 | 3,53 | 3,92 | 2.89% |  |  |  |  |  |
| Kumar 2006 | 11,78 | 11,20 | 12,37 | 2.88% |  |  |  |  |  |
| Bundrant 2011 | 10,79 | 9,73 | 11,86 | 2.86% |  |  |  |  |  |
| Loken 1982.1 | 0,03 | -0,77 | 0,83 | 2.87% |  |  |  |  |  |
| Ali 2024.1 | 13,45 | 10,70 | 16,19 | 2.66% |  |  |  |  |  |
| Ali 2024.2 | 8,40 | 6,85 | 9,94 | 2.82% |  |  |  |  |  |
| Ali 2024.3 | 2,19 | 1,06 | 3,33 | 2.85% |  |  |  |  |  |
| Ali 2024.4 | 13,90 | 12,00 | 15,80 | 2.77% |  |  |  |  |  |
| Ali 2024.5 | 11,25 | 8,42 | 14,09 | 2.65% |  |  |  |  |  |
| Ali 2024.6 | 6,35 | 4,35 | 8,36 | 2.77% |  |  |  |  |  |
| Ali 2024.7 | 13,82 | 11,12 | 16,52 | 2.67% |  |  |  |  |  |
| Ali 2024.8 | 8,22 | 4,01 | 12,42 | 2.49% |  |  |  |  |  |
| Prado 2019 | 13,26 | 10,34 | 16,17 | 2,64% |  |  |  |  |  |
| **CNS** | 9,46 | 7,68 | 11,25 | 25,47% | 99,43% | 21,41 | 4,63 | -0,211 | 19,04 |
| Otter 2004.1 | 8,79 | 7,82 | 9,75 | 12,36% |  |  |  |  |  |
| Otter 2004.2 | 3,30 | 2,64 | 3,96 | 12,68% |  |  |  |  |  |
| Otter 2004.3 | 1,84 | 1,31 | 2,38 | 12,78% |  |  |  |  |  |
| Otter 2004.4 | 1,38 | 1,02 | 1,74 | 12,88% |  |  |  |  |  |
| Otter 2004.5 | 2,14 | 1,13 | 3,15 | 12,31% |  |  |  |  |  |
| Otter 2004.6 | 9,07 | 7,85 | 10,30 | 12,02% |  |  |  |  |  |
| Fairley 2013 | 2,83 | 1,64 | 4,03 | 12,07% |  |  |  |  |  |
| Garcia 2016 | 1,15 | 0,79 | 1,50 | 12,89% |  |  |  |  |  |
| **Diarrhoea** | **3,76** | **1,53** | **5,99** | **25,22%** | **98,10%** | **4,88** | **2,21** | **-2,11** | **9,64** |
| Gitter 1965.1 | 2,79 | 2,45 | 3,12 | 50,20% |  |  |  |  |  |
| Loken 1982.2 | -0,93 | -1,51 | -0,35 | 49,80% |  |  |  |  |  |
| **Septicemia** | **0,94** | **-2,72** | **4,59** | **24,04%** | **99,19%** | **6,86** | **2,62** | **-39,87** | **41,74** |
| **Combined Effect Size** | **2,40** | **1,25** | **3,55** |  | **99,66%** | **30,63** | **5,53** | **-10,75** | **15,55** |
